# Serological and Molecular Epidemiology of Hepatitis B, C, and D Viruses in Northwest Russia: A Population-Based Cross-Sectional Study

**DOI:** 10.3390/v18060632

**Published:** 2026-05-30

**Authors:** Anna Y. Popova, Yulia V. Ostankova, Alesia Y. Olkhovskaya, Olga A. Petrova, Alexandr N. Shchemelev, Elena N. Serikova, Svetlana A. Egorova, Diana E. Reingardt, Irina V. Drozd, Ojuna B. Zhimbaeva, Ekaterina M. Danilova, Angelica M. Milichkina, Elena B. Ezhlova, Albina A. Melnikova, Natalia S. Bashketova, Lidiya V. Buts, Edward S. Ramsay, Areg A. Totolian

**Affiliations:** 1 Federal Service for the Oversight of Consumer Protection and Welfare, 127994 Moscow, Russiaezhlova@pasteurorg.ru (E.B.E.); melnikova@pasteurorg.ru (A.A.M.); 2 Saint Petersburg Pasteur Institute, 197101 St. Petersburg, Russia; shenna1@yandex.ru (Y.V.O.); alesja.gorbynova@gmail.com (A.Y.O.); belka-mbf1988@mail.ru (O.A.P.); genista.bio@gmail.com (E.N.S.); egorova@pasteurorg.ru (S.A.E.); dianavalutite008@gmail.com (D.E.R.); div-o@mail.ru (I.V.D.); zhimbaeva@pasteurorg.ru (O.B.Z.); emdanilova@pasteurorg.ru (E.M.D.); amilichkina@pasteurorg.ru (A.M.M.); butz@pasteurorg.ru (L.V.B.); totolian@spbraaci.ru (A.A.T.); 3 Federal Service for the Oversight of Consumer Protection and Welfare, 192029 St. Petersburg and Leningrad Region, Russia; uprav@78rospotrebnadzor.ru

**Keywords:** hepatitis B virus (HBV), hepatitis C virus (HCV), hepatitis delta virus (HDV), occult HBV infection (OBI), seroprevalence, molecular epidemiology, vaccination coverage, iatrogenic transmission, vaccine-induced immunity, Russia

## Abstract

The hepatitis B (HBV), C (HCV), and D (HDV) viruses remain a major public health burden. Occult HBV infection (OBI) represents a hidden reservoir with clinical and epidemiological significance, yet its prevalence in Northwest Russia is unknown. We aimed to comprehensively assess the serological and molecular epidemiology of HBV, HCV, and HDV in St. Petersburg and the Leningrad region. **Methods.** In this cross-sectional study, 6773 apparently healthy volunteers were enrolled. Plasma samples were tested for hepatitis B surface antigen (HBsAg), antibodies to HBV core antigen (anti-HBc), antibodies to HBsAg (anti-HBs), antibodies to HCV (anti-HCV), and antibodies to HDV (anti-HDV) by ELISA. All anti-HCV- and anti-HDV-positive samples were tested for HCV RNA and HDV RNA by real-time PCR. All samples were tested for HBV DNA using a highly sensitive in-house nested real-time PCR assay (detection limit: 5 IU/mL). All “HBV DNA-positive, HBsAg-negative” cases confirmed by two independent extractions were classified as OBI. Vaccination status, self-reported history, and iatrogenic interventions were recorded. **Results.** Overall seroprevalence values were: HBsAg 1.7%; anti-HBc 11.3%; anti-HBs 43.0%; anti-HCV 1.9%; and anti-HDV 0.6%. Anti-HBc increased sharply with age (3.1% in children to 26.4% in the elderly, *p* < 0.0001), while anti-HBs declined (69.9% to 29.8%, *p* < 0.0001). HBV DNA was detected in 118 participants (1.7%). Of these, only 73 individuals (1.1%) were HBsAg-positive, while the remaining 45 participants (0.7%) had undetectable HBsAg, meeting the criteria for OBI. OBI was detected across all age groups, including children. Serological profiling of OBI cases revealed that 57.8% lacked both anti-HBc and anti-HBs, 35.6% had isolated anti-HBs, 2.2% had isolated anti-HBc, and 4.4% had both antibodies. HCV RNA was detected in 15.0% of anti-HCV-positive individuals (all adults). No HDV RNA was detected. Self-reported history underestimated true infection rates: 1.4% of those denying HBV infection were HBsAg-positive and 10.6% were anti-HBc-positive. Among those denying HCV infection, 1.4% were anti-HCV-positive. Vaccination coverage was 70.8%, declining from 90.9% in children to 39.0% in the elderly (*p* < 0.0001). Among vaccinated individuals, 48.0% lacked protective anti-HBs (<10.0 mIU/mL). **Conclusions.** This comprehensive serological and molecular study in Northwest Russia is the first to combine population-level serology with molecular detection of HBV, HCV, and HDV, including OBI in this region, and reveals that OBI accounts for a substantial proportion (38%) of all active HBV infections and is strongly associated with a history of iatrogenic interventions. The presence of OBI across all age groups, including children, shows that HBsAg screening alone substantially underestimates the true HBV burden. High rates of unrecognized infection and waning vaccine-induced immunity, highlight critical gaps in current surveillance. These findings provide an evidence-based rationale for integrating molecular testing into screening algorithms and for considering booster vaccination strategies to achieve viral hepatitis elimination goals.

## 1. Introduction

Viral hepatitis B (HB), C (HC), and D (HD) are caused by hepatotropic viruses with distinct biological characteristics. They remain a global public health concern due to their high morbidity, mortality, and significant socioeconomic consequences [1]. Despite their differences, these infections share the ability to cause chronic liver damage, leading to cirrhosis and hepatocellular carcinoma (HCC) [2,3]. Hepatitis B virus (HBV) and hepatitis C virus (HCV) can cause acute disease, ranging from asymptomatic to severe [4,5,6,7]. However, their outcomes differ. Only about 5–10% of adults with acute HBV infection develop chronic infection. With HCV, this figure is 50–80% [5,6]. Notably, the risk of HBV chronification reaches 95% in cases of perinatal transmission [8,9]. HBV chronically infects approximately 257–296 million people, and HCV about 58 million [10]. The global prevalence of hepatitis D virus (HDV) is estimated at around 13% among HBsAg-positive individuals. However, the true scale may be underestimated, especially in regions without routine anti-HDV screening [11,12,13].

The epidemiological situation regarding parenteral viral hepatitis globally, and specifically in Russia, is characterized by high variability. It is influenced by socioeconomic factors, migration dynamics, vaccination programs, and access to therapy [14,15,16,17,18,19,20]. The introduction of highly effective antiviral drugs can significantly reduce morbidity, mortality, and economic burden [21,22,23]. Yet, the curative potential differs between hepatitis C and B: direct-acting antivirals (DAAs) achieve >95% sustained virologic response in HCV, whereas HBV therapy suppresses viral replication but does not eliminate the nuclear covalently closed circular DNA (cccDNA) reservoir. However, low public awareness and underdiagnosis significantly worsen the overall situation, insofar as a substantial proportion of infected individuals do not receive treatment, leading to a persistently high overall disease burden [23,24,25,26,27].

From a biological and diagnostic standpoint, a key challenge in controlling HBV remains occult hepatitis B infection (OBI) [28,29]. OBI is characterized by the presence of replication-competent HBV DNA in the blood or liver tissue in the absence of the primary serological marker of infection, hepatitis B surface antigen (HBsAg) [30]. This phenomenon creates a hidden reservoir of infection with significant clinical and epidemiological importance. OBI is associated with the risk of viral reactivation during immunosuppression [31,32]. It may contribute to the progression of liver fibrosis and the development of HCC, and it can influence treatment outcomes for concomitant diseases [33,34,35]. Its epidemiological significance [36] is further compounded by the extremely low infectious dose of HBV, which elevates the risk of transmission, particularly during iatrogenic interventions [37]. The most effective primary preventative measure for HB is vaccination, which induces specific humoral immunity (anti-HBs) [38,39]. The prevalence of OBI in populations with different levels of HBV vaccination coverage, particularly in regions with transitioning epidemiology, remains insufficiently studied [40,41].

In St. Petersburg and Leningrad Oblast, no large-scale study has yet combined serological and molecular testing to comprehensively assess parenteral viral hepatitis, including occult HBV infection and HDV co-infection.

In this study, we aimed to conduct a comprehensive epidemiological assessment of the local (St. Petersburg, Leningrad Oblast) population at the serological and molecular level. We analyzed prevalence and interrelationships among the viruses (hepatitis B/C/D) and their markers using an array of diagnostics. These included serological markers and viral nucleic acid analyses. Age, activity/profession, infectious status, vaccination status (HBV), and iatrogenic intervention history were also taken into account.

## 2. Materials and Methods

### 2.1. Study Design and Ethics Approval

A cross-sectional randomized study was conducted as part of the Rospotrebnadzor (Russian Federal Service for the Oversight of Consumer Protection and Welfare) program “Assessment of herd immunity to vaccine-preventable and other significant infections in the population of St. Petersburg and the Leningrad region”. The study protocol was approved by the Local Ethics Committee of the St. Petersburg Pasteur Institute of Epidemiology and Microbiology (Protocol No. 88, dated 3 October 2023). All participants, or their legal representatives, were informed about the purpose and methodology of the study and provided written informed consent.

### 2.2. Study Population and Sampling

Volunteers were recruited through a questionnaire. The questionnaire collected the following personalized data: full name; gender; date of birth; address; occupation; attendance (preschools, schools, universities); contact details; healthcare facility of registration; chronic diseases; blood transfusions; surgical interventions; and history related to parenterally transmitted viral hepatitis (previous infection, HBV vaccination). Information on past infection and vaccination was verified using vaccination certificates or other medical records.

Following questionnaire processing, volunteers meeting the inclusion/exclusion criteria were invited to the St. Petersburg Pasteur Institute medical center for subsequent laboratory testing. Inclusion criteria were: individuals aged 1 year or older; residence in St. Petersburg or the Leningrad region; and providing informed consent. Exclusion criteria were: refusal to undergo venipuncture for blood sample collection and/or active form of any infectious disease at the time of sampling.

The sample size for a representative population was calculated using a formula based on the De Moivre-Laplace limit theorem, as described previously [42,43,44], utilizing an online calculator [45]. The final cohort consisted of 6773 conditionally healthy volunteers from St. Petersburg (*n* = 3300) and the Leningrad region (*n* = 3473). Volunteers were stratified into nine age groups in years: 1–5 (*n* = 370), 6–11 (*n* = 511), 12–17 (*n* = 538), 18–29 (*n* = 792), 30–39 (*n* = 838), 40–49 (*n* = 914), 50–59 (*n* = 900), 60–69 (*n* = 930), and ≥70 years (*n* = 980). The proportions of adult age groups in the total sample were comparable, differing by 1.5–2.5%. Volunteers were recruited from all 18 districts of St. Petersburg and 17 districts of the Leningrad region. The cohort comprised 26.4% males (*n* = 1789) and 73.6% females (*n* = 4984) [42,43,44].

### 2.3. Specimen Collection

Whole blood samples were collected into EDTA-containing vacuum tubes. Plasma was separated by centrifugation (1500–2000× *g* for 10 min.) and stored at −20 °C until analysis.

### 2.4. Serological Testing

Plasma samples were screened for serological markers (HB, HC, HD). Enzyme-linked immunosorbent assays (ELISA) were used to detect HB surface antigen (HBsAg), total Abs to HBV core antigen (anti-HBc), Abs to HBsAg (anti-HBs), Abs to HCV (anti-HCV), and Abs to HDV (anti-HDV). To assess the humoral immune response to HBV, the presence and quantitative levels of anti-HBs Abs were determined. All screening assays were performed using commercial ELISA kits from Diagnostic Systems (Nizhny Novgorod, Russia) in strict accordance with the manufacturer’s protocols: “DS-ELISA-HBsAg” for HBsAg (analytical sensitivity: 0.01 IU/mL); “DS-ELISA-ANTI-HBc” for total anti-HBc; “DS-ELISA-ANTI-HBsAg” for quantitative anti-HBs; “ELISA-ANTI-HCV” for anti-HCV; and “ELISA-ANTI-HDV” for anti-HDV. Initial reactive or borderline results for HBsAg and anti-HCV were subjected to confirmatory testing. The “ELISA-HBsAg-confirmatory test” (Diagnostic Systems) was used for HBsAg neutralization. The “DS-ELISA-ANTI-HCV-SPECTR-GM” (Diagnostic Systems) was employed for supplemental anti-HCV testing.

### 2.5. Molecular Detection of Viral Nucleic Acids

Nucleic acids were extracted from 200 µL aliquots of plasma using the commercial kit “NK-Magno-UltraPure-A” (Russia) according to the manufacturer’s instructions.

#### 2.5.1. HBV DNA Detection and OBI Confirmation

HBV DNA was tested using a highly sensitive in-house-developed nested real-time PCR assay with hybridization-fluorescence detection. It targets three conserved regions of the HBV genome (core, S, and X genes) and includes an endogenous internal control (human HPRT gene) [46]. The analytical sensitivity of the method is 5 IU/mL when DNA is extracted from 200 µL of plasma. Quantitative assessment of viral load was not performed insofar as commercially available quantitative kits typically have a higher detection threshold (50–100 IU/mL) and may underestimate the true prevalence of occult infections.

For all samples with detectable HBV DNA, the result was confirmed by a second, independent extraction performed from a reserved, previously unprocessed aliquot of the same plasma sample. This procedure reduced the risk of false-negative results and ensures robust OBI case classification. A case was defined as OBI when HBV DNA was detected in at least two independent extractions in the absence of HBsAg.

#### 2.5.2. HCV and HDV RNA Detection

Samples seropositive for anti-HCV or anti-HDV were subsequently tested for HCV RNA or HDV RNA, respectively. The commercial real-time PCR kits “AmpliSens HCV-FL” or “AmpliSens HDV-FL” were used (Central Research Institute of Epidemiology, Moscow, Russia). The analytical sensitivity of both assays is 100 IU/mL when nucleic acids are extracted from 100 µL of plasma. All PCR assays were performed following the manufacturer’s protocols.

### 2.6. Statistical Analysis

Data organization was performed using Microsoft Excel (Microsoft Corporation, Redmond, WA, USA). Statistical analyses were conducted using GraphPad Prism version 9.3.0 (GraphPad Software, San Diego, CA, USA). Prevalence estimates are presented with 95% confidence intervals (95% CIs) calculated using the Clopper–Pearson exact method. Proportions between two independent groups were compared using Fisher’s exact test or Yates’ corrected Chi-square test, as appropriate. The Cochran–Armitage test for linear trend was applied to assess the statistical significance of age-related trends in marker prevalence, using the mean age of each interval group as the independent variable. The strength and direction of the monotonic association between age and marker prevalence were assessed using Spearman’s rank correlation coefficient (rs). Differences and correlations were considered statistically significant at a *p*-value < 0.05.

## 3. Results

### 3.1. Demographic and Clinical Characteristics

**Figure 1:**
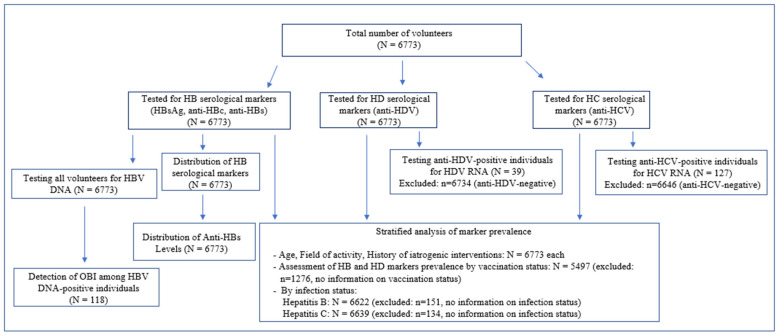
Flowchart of viral marker analyses and analytical subgroups. The demographic and clinical characteristics of the cohort are presented here to provide context for subsequent analyses. The broad age distribution was as follows: children and adolescents (1–17 years, *n* = 1415), working-age adults (18–69 years, *n* = 4378), and elderly participants (70+ years, *n* = 980). The cohort included 1366 healthcare workers and 592 educators, which are key groups for assessing occupational risk. Healthcare workers (“Medicine”) included physicians, nurses, and laboratory personnel with occupational exposure to patients or biological fluids. Educators (“Education”) include teachers in schools, colleges, vocational schools, as well as caregivers in kindergartens and preschool educational institutions. A history of iatrogenic intervention (surgery, blood transfusion) was reported by 2632 (38.9%) participants. After excluding participants unsure of their status, 6622 individuals (97.8%) provided a definitive response regarding HB. Among these respondents, a history of HB was self-reported by 63 participants, yielding a prevalence of 0.95% in this subgroup. For HC, 6639 participants (98.0%) were certain of their status, and 29 (0.4%) reported a positive history. Regarding HB vaccination, 5497 volunteers (81.2%) were certain of their status. Within this group, 3890 participants (70.8%) reported having been vaccinated.

The study population consisted of 6773 volunteers from St. Petersburg and the Leningrad region. The selection of participant samples for each type of analysis (workflow) is presented visually in Figure 1.

### 3.2. Seroprevalence of HB, HC, and HD Markers

**Figure 2:**
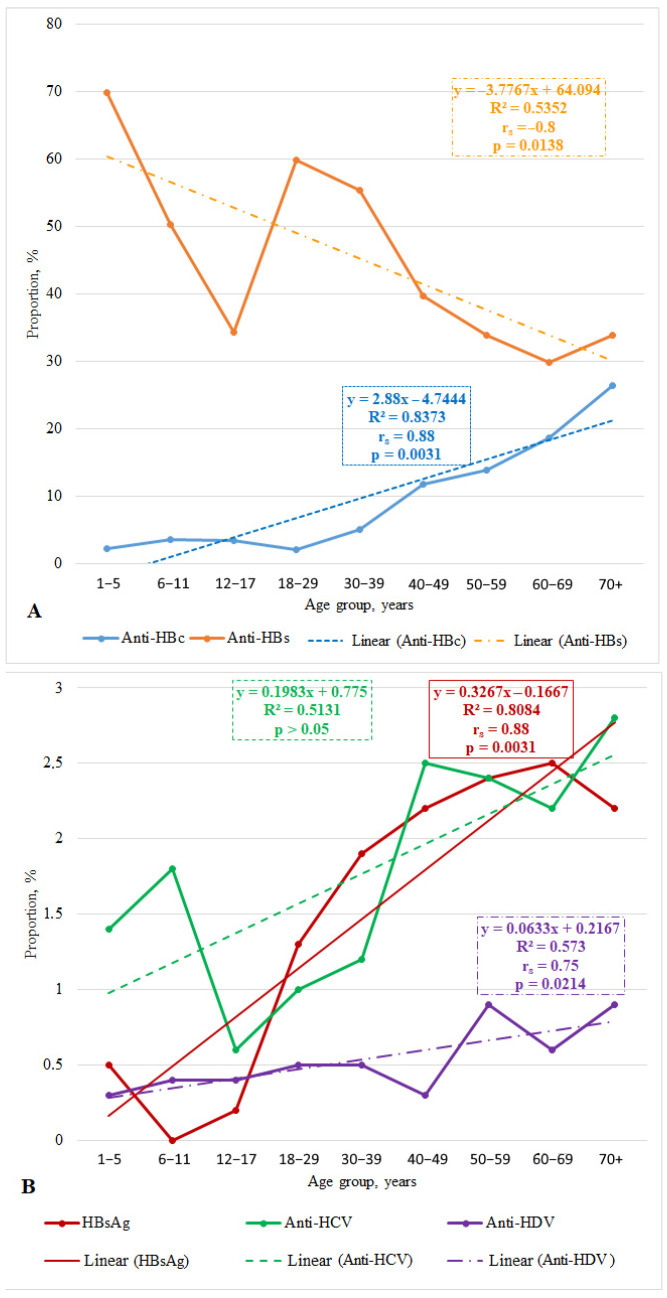
Correlation assessment between marker frequency and volunteer age. (**A**) Anti-HBs and anti-HBcore markers. (**B**) HBsAg, anti-HCV, and anti-HDV markers. Regression equations, coefficients of determination (R^2^), Spearman’s correlation coefficients (rs), and statistical significance values (*p*) are presented (color-coded according to trend).

Among the entire cohort (N = 6773), HBsAg was detected in 116 individuals (1.7%), anti-HBc in 768 (11.3%), anti-HBs in 2915 (43.0%), anti-HCV in 127 (1.9%), and anti-HDV in 39 participants (0.6%) (Table 1). The prevalence of all markers, except anti-HDV, showed a strong age dependence (Table 1, Figure 2). The prevalence of anti-HBc, a marker of past or current HBV infection, increased progressively with age, from 3.1% in the 1–17 years group to 26.4% in participants aged 70+ (χ^2^ for trend = 404.28, *p* < 0.0001). The most pronounced increase occurred between the 30–39 and 40–49 age groups (from 5.0% to 11.8%, χ^2^ = 24.92, *p* < 0.001). HBsAg prevalence was 1.7% overall, with only three cases detected among children and adolescents aged 1–17 years (0.2%). Values increased progressively with age, reaching an average of 2.1% in adults and peaking at 2.5% in the 60–69 age group. The Cochran–Armitage test for linear trend confirmed a significant increase in HBsAg prevalence with age (χ^2^ for trend = 23.107, *p* < 0.0001). The difference between children and adults was also significant (χ^2^ = 22.81, *p* < 0.0001).

In contrast, the prevalence of anti-HBs (which indicates vaccine- or infection-induced immunity) was highest in the youngest children (69.9% in the 1–5 years group) and progressively decreased with age. A significant decline was observed between the 1–5 year- and 12–17-year age groups (34.3%, χ^2^ = 109.37, *p* < 0.0001). Among adults, prevalence continued to decrease gradually, reaching its minimum of 29.8% in the 60–69 years group (χ^2^ for trend among adults = 277.97, *p* < 0.0001).

Anti-HCV prevalence was significantly higher in adults compared to children and adolescents (χ^2^ = 3.96, *p* = 0.046). The Cochran–Armitage test for linear trend confirmed a significant increase in anti-HCV prevalence with age (χ^2^ for trend = 10.977, *p* = 0.0009). Anti-HDV prevalence was low (0.6%) and sporadic across all age groups, with no clear age trend (*p* > 0.05).

In the tables throughout this article, asterisks (*) and hash marks (#) are used to indicate subgroups with prevalence estimates that are significantly higher (*) or lower (#) than the overall prevalence in the total cohort (the “Total” row). This approach, commonly employed in population-based epidemiological studies, allows readers to quickly identify subgroups that deviate from the cohort average, providing a valuable descriptive overview of the data. The rationale for such comparisons is grounded in the principle that “the overall disease metric for any population is a direct reflection of the burden among subgroups or subpopulations”. Tracking subgroup-specific burden is essential for assessing the impact of public health interventions, particularly for infectious diseases where intervention effectiveness is often dependent on targeting specific subgroups [47].

Spearman correlation analysis confirmed these age-related trends (Figure 2). A strong positive correlation with age was found for anti-HBc (rs = 0.88, *p* = 0.0031), HBsAg (rs = 0.88, *p* = 0.0031), and anti-HDV (rs = 0.75, *p* = 0.0214). A strong negative correlation was observed for anti-HBs (rs = −0.8, *p* = 0.0138). For anti-HCV, the correlation did not reach significance (rs = 0.68, *p* = 0.0503). This is likely due to the non-linear pattern of prevalence across age groups, with detectable anti-HCV levels in children (1.2%) and young adults (1.0%) contributing to deviation from a strictly monotonic increase, despite the significant linear trend identified by the Cochran–Armitage test. Due to the substantial differences in prevalence values between the highly prevalent markers (anti-HBs, anti-HBc) and the rare ones (HBsAg, anti-HCV, anti-HDV), the data are presented on two separate graphs with different *Y*-axis scales for clarity (Figure 2A,B).

### 3.3. Analysis of HBV Serological Profiles

The distribution of HB serological profiles (HBsAg/anti-HBc/anti-HBs) in the studied cohort is presented in Table 2. These profiles allow tentative differentiation between vaccine-induced immunity, past resolved infection, and current active infection. The most common profile was ‘anti-HBs only’, observed in 2320 participants (34.3%). While this pattern is highly suggestive of vaccine-induced immunity, it may also occur in individuals after resolved infection with loss of anti-HBc over time. Markers indicative of past resolved HBV infection (anti-HBc- and anti-HBs-positive) were found in 560 individuals (8.3%). Anti-HBc only, a profile that may represent resolved infection with loss of anti-HB or potentially occult infection, was present in 167 participants (2.5%).

Profiles indicating current or possible active HBV infection (HBsAg-positive) included ‘HBsAg only’ (0.8%, *n* = 51), ‘HBsAg and anti-HBc’ (0.4%, *n* = 30), ‘HBsAg and anti-HBs’ (0.4%, *n* = 26), and the simultaneous presence of all three markers (0.1%, *n* = 9). Overall, at least one HBV marker was detected in 3163 individuals (46.7%), while 3610 participants (53.3%) were seronegative for all HBV markers.

### 3.4. Seroprevalence in Key Activity and Demographic Groups

**Figure 3:**
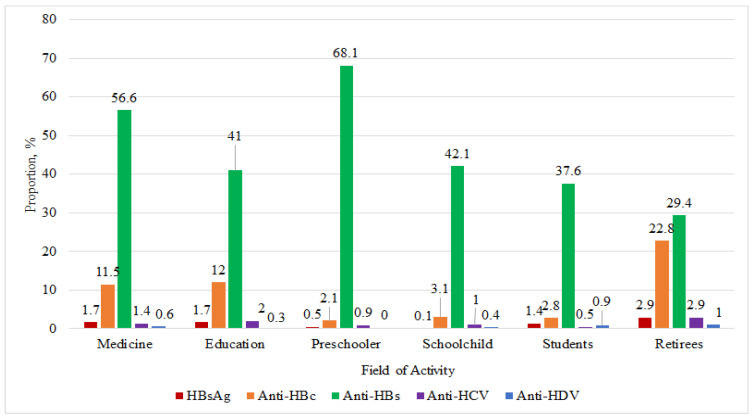
Hepatitis B/C/D markers in epidemiologically significant groups.

Hepatitis marker prevalence varied significantly across different professional and social groups (Figure 3). Detailed data for all categories are provided in Table A1 (Appendix A).

The highest frequency of anti-HBc, indicating exposure to HBV, was observed among retirees (22.8%), reflecting cumulative risk with age, as well as among unemployed individuals (17.1%) and those working in production (14.9%). In contrast, the lowest anti-HBc prevalence was found in preschoolers (2.1%) and schoolchildren (3.1%).

The prevalence of anti-HBs (a marker of vaccine- or infection-induced immunity) was highest in groups covered by routine childhood immunization: preschoolers (68.1%) and schoolchildren (42.1%). Among occupational groups, healthcare workers showed the highest anti-HBs prevalence (56.6%), significantly exceeding that of educators (41.0%, χ^2^ = 39.34, *p* < 0.0001). The lowest anti-HBs prevalence was observed among retirees (29.4%).

We further analyzed the distribution of the seven HBV serological profiles across the six key activity groups shown in Figure 3. The complete data are presented in Appendix A (Table A2). In brief, anti-HBs only was significantly more frequent in healthcare workers (45.9%) than in educators (31.3%; χ^2^ = 35.92, *p* < 0.0001) and was highest in preschoolers (66.5%). Isolated anti-HBc was rare overall but was more common in retirees (5.8%). Notably, the combination of HBsAg with anti-HBs was absent in schoolchildren; HBsAg with anti-HBc and the triple-positive profile were absent in preschoolers, schoolchildren, and students. The combination of HBsAg with anti-HBc was significantly more frequent in retirees (1.0%) than in healthcare workers (0.1%; χ^2^ = 7.47, *p* = 0.0063).

Anti-HCV positivity was most frequently detected in retirees (2.9%), unemployed individuals (3.6%), and those in the “miscellaneous” category (4.0%). Its prevalence in healthcare workers (1.4%) and educators (2.0%) was close to the cohort average (1.9%). Anti-HDV prevalence was low across all groups, with the highest estimates observed in office workers (1.6%) and business professionals (1.3%), although confidence intervals were wide due to small case numbers.

### 3.5. Association with Iatrogenic Interventions

The association between a history of iatrogenic interventions (surgery, blood transfusion) and the seroprevalence of viral hepatitis markers was analyzed (Table 3). Statistically significant differences were observed between groups with and without such a history. Participants reporting a history of such interventions had a significantly higher prevalence of anti-HBc compared to those without such interventions (13.8% vs. 9.8%, χ^2^ = 26.16, *p* < 0.0001, OR = 1.48, 95% CI: 1.3–1.7). Conversely, the prevalence of anti-HBs was significantly lower in the group with iatrogenic interventions (40.0% vs. 44.9%, χ^2^ = 15.5, *p* < 0.0001, OR = 0.8, 95% CI: 0.7–0.9).

For anti-HCV and anti-HDV, a trend toward higher prevalence in those with a history of iatrogenic interventions did not reach significance (*p* > 0.05). In contrast, HBsAg was significantly more frequent in individuals without such interventions (2.1% vs. 1.2%; χ^2^ = 6.8, *p* = 0.009), while the opposite pattern was observed for anti-HBc. This discrepancy is explored further in Section 3.9 in the context of occult HBV infection.

We further analyzed the distribution of the seven HBV serological profiles according to a history of iatrogenic interventions (surgery or blood transfusion). The complete data are presented in Appendix A (Table A3). In brief, the frequency of isolated anti-HBs was significantly higher in individuals without such interventions (37.4%) compared to those with a history of interventions (29.3%; χ^2^ = 45.95, *p* < 0.0001). Conversely, the combination of anti-HBs and anti-HBc was more common in the intervention group (10.5% vs. 6.9%; χ^2^ = 27.45, *p* < 0.0001). No significant differences were observed for the other serological profiles.

### 3.6. Population Immunity to HBV: Distribution of Anti-HBs Levels

**Figure 4:**
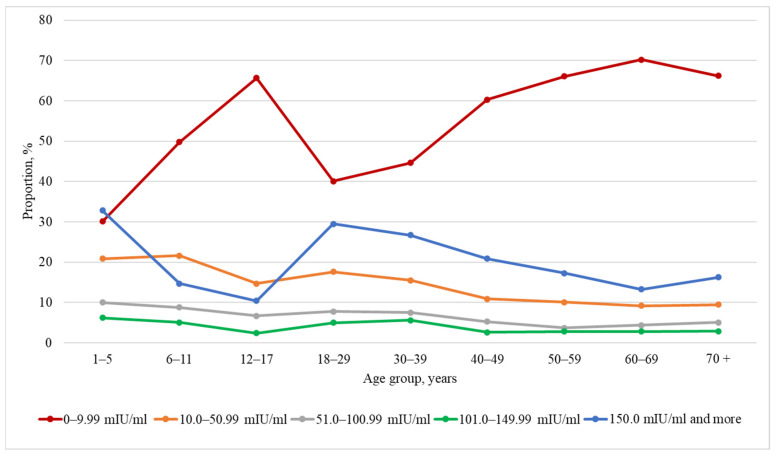
Distribution of anti-HBs levels by age group.

**Figure A1:**
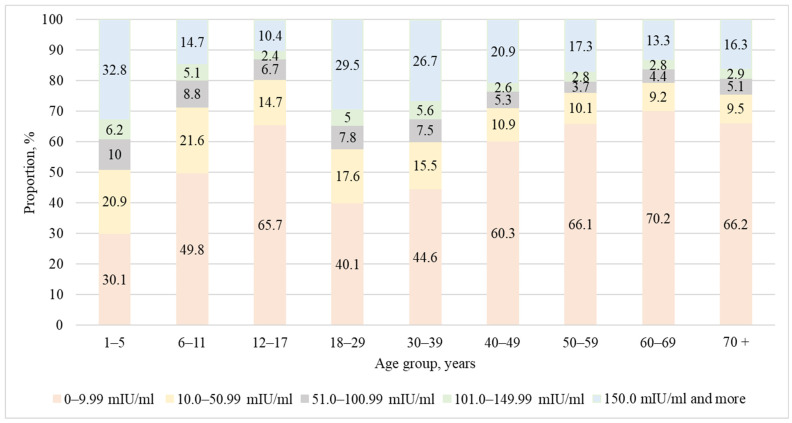
Distribution of anti-HBs levels among volunteers by age group. Numerical values are provided in Table A3.

The distribution of anti-HBs levels across different age groups was analyzed to assess the actual status of serological protection in the population, regardless of the source of immunity (vaccination or past infection). Age-related trends in anti-HBs levels are shown in Figure 4. A detailed breakdown of the structural composition of immunity by age group is provided in Figure A1 (Appendix A), along with the corresponding numerical data in Table A4.

Overall, seroprotective anti-HBs levels (≥10.0 mIU/mL) were detected in 2915 participants, accounting for 43.0% of the total cohort (N = 6773). High Ab levels (≥150.0 mIU/mL), indicating a robust immune response, were observed in 1342 individuals (19.8%). Conversely, 3858 participants (57.0%) had anti-HBs levels below the protective threshold (<10.0 mIU/mL).

Marked age-related differences in the distribution of anti-HBs levels were observed. The highest share of individuals with high Ab levels (≥150.0 mIU/mL) was found in the youngest children (32.8% in the 1–5 years group, 95% CI: 28.2–37.7) and in young adults (29.5% in the 18–29 years group, 95% CI: 26.5–32.8). A secondary peak was also observed in the 30–39-year age group (26.7%, 95% CI: 23.8–29.8).

In contrast, the prevalence of seronegative individuals (anti-HBs <10.0 mIU/mL) increased with age, reaching its maximum in the older age groups. The highest shares of seronegative participants were observed in the 60–69 years group (70.2%, 95% CI: 67.2–73.1) and among those aged 70+ (66.2%, 95% CI: 63.2–69.1). A notable increase in seronegativity was also observed during adolescence, rising from 30.1% in the 1–5 years group to 65.7% in the 13–17 years group. Intermediate anti-HBs levels (10.0–149.9 mIU/mL) were more uniformly distributed across age groups, collectively accounting for 23.2% of the total cohort (*n* = 1573).

### 3.7. HBV Vaccination Status and Its Association with Serological Markers

HBV vaccination status was analyzed in the 5497 participants who were certain of their history. Of these, 3890 (70.8%) reported being vaccinated against HBV, while 1607 (29.2%) reported no vaccination (Table 4).

**Figure 5:**
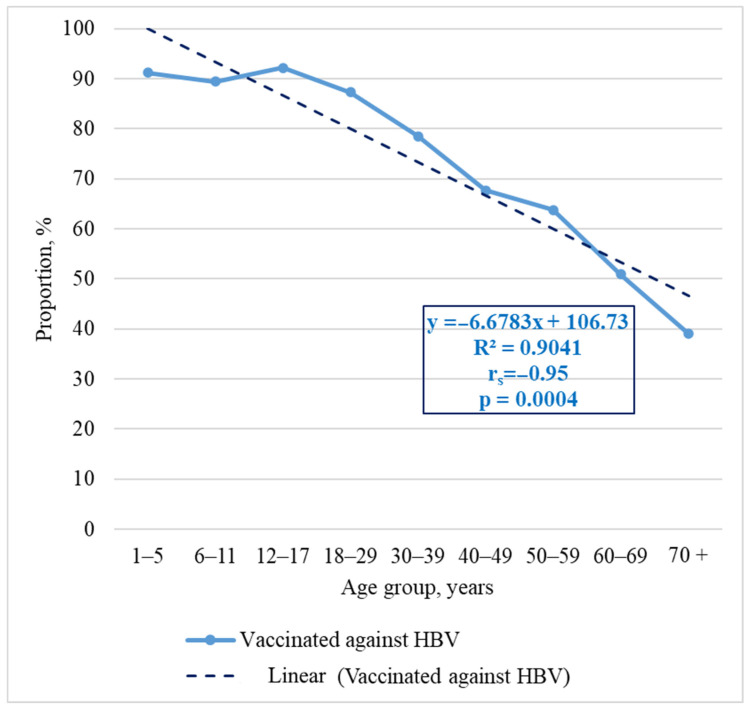
Correlation between share vaccinated and age group. The regression equation, coefficient of determination (R^2^), Spearman’s correlation coefficient (r_s_), and statistical significance values (*p*) are shown.

The highest vaccination coverage was observed in the 1–17-year age group (90.9%, *n* = 1209/1330), with no significant differences between pediatric subgroups. A progressive decline in coverage was observed with increasing age, from 87.3% in the 18–29 years group to 39.0% in participants aged 70+ (χ^2^ for trend = 463.04, *p* < 0.0001). Spearman correlation analysis confirmed a strong negative association between vaccination coverage and age (rs = −0.95, *p* = 0.0004), with age explaining over 90% of the variance (R^2^ = 0.904) (Figure 5).

**Figure 6:**
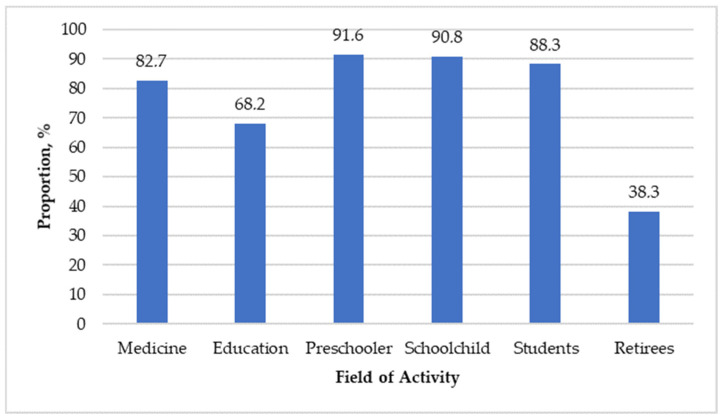
Vaccination coverage (hepatitis B) by volunteer activity.

Analysis of vaccination coverage by professional/social group showed the highest values among those covered by routine immunization programs: preschoolers (91.6%), schoolchildren (90.8%), and students (88.3%). The pattern is shown in Figure 6. Detailed data are given in Table A5 (Appendix A). Among occupational groups, healthcare workers had significantly higher coverage (82.7%) compared to educators (68.2%, χ^2^ = 41.35, *p* < 0.0001). The lowest coverage was observed in retirees (38.3%).

Comparison of HBV and HDV markers between vaccinated and unvaccinated individuals revealed significant differences (Table 5). Vaccinated participants had a significantly higher prevalence of anti-HBs (52.2% vs. 26.9%, χ^2^ = 292.5, *p* < 0.0001) and significantly lower prevalence of anti-HBc (8.7% vs. 15.4%, χ^2^ = 52.6, *p* < 0.0001) and HBsAg (1.3% vs. 2.2%, χ^2^ = 5.4, *p* = 0.0204, OR = 1.7, 95% CI: 1.1–2.6). No significant difference was observed for anti-HDV prevalence.

A more detailed analysis of anti-HBs levels by vaccination status showed that vaccinated individuals not only had a higher frequency of seroprotection, but also higher Ab concentrations (Table 6). Among vaccinated participants, 23.8% had high Ab levels (≥150.0 mIU/mL), compared to only 12.3% among unvaccinated individuals (χ^2^ = 91.8, *p* < 0.0001). Conversely, the share of seronegative individuals (anti-HBs <10.0 mIU/mL) was significantly lower in the vaccinated group (48.0%) than in the unvaccinated group (73.0%, χ^2^ = 292.5, *p* < 0.0001).

To further elucidate the interplay between vaccination and natural infection, participants who were certain of both their vaccination and infectious history (N = 5427) were categorized into four groups: “history-positive, not vaccinated” (HPNV, *n* = 34); “history-positive, vaccinated” (HPV, *n* = 16); “history-negative, not vaccinated” (HNNV, “naive”, *n* = 1538); and “history-negative, vaccinated” (HNV, *n* = 3839). These distinctions are shown in Table 7. They are also detailed as age-stratified data in Table A6, Table A7, Table A8 and Table A9 (Appendix A).

As expected, markers of infection (HBsAg, anti-HBc) were most prevalent in groups reporting a history of hepatitis. Anti-HBs was most frequently detected in the vaccinated-only group (52.4%), followed by the infected-only group (35.3%), and was lowest in the naive group (27.0%). Notably, in the naive group (those denying both infection and vaccination), anti-HBc was detected in 14.0% and HBsAg in 1.4%, indicating unrecognized past or current infection. In the vaccinated-only group, the prevalence of anti-HBc was lower (8.4%).

The full distribution of the seven HBV serological profiles across the four groups (HPNV, HPV, HNNV, and HNV) is presented in Appendix A (Table A10). Several distinct patterns emerged. First, profiles containing HBsAg together with anti-HBs (with or without anti-HBc) were absent in both groups with a self-reported history of hepatitis B (HPNV and HPV), but were present at low frequencies in the vaccinated-only group (HNV: HBsAg+anti-HBs 0.4%, triple-positive 0.1%). Second, isolated anti-HBs was significantly more frequent in HNV (45.1%) than in HNNV (16.3%; χ^2^ = 390.54, *p* < 0.0001), consistent with vaccine-induced immunity. Conversely, isolated anti-HBc was significantly more common in HPNV (17.6%) compared to HNNV (3.6%; χ^2^ = 14.08, *p* = 0.0002) and HNV (1.5%; χ^2^ = 46.26, *p* < 0.0001), reflecting resolved natural infection. Third, the combination of anti-HBs and anti-HBc was significantly more frequent in the groups with a history of hepatitis B (HPNV 35.3%, HPV 43.8%) than in those without (HNNV 9.8%, HNV 6.7%). Among the non-infected groups, this combination was less frequent in vaccinated individuals (HNV 6.7%) than in unvaccinated individuals (HNNV 9.8%; χ^2^ = 15.12, *p* = 0.0001).

The prevalence of HBV DNA, including OBI, in these groups is analyzed in Section 3.9.

### 3.8. Molecular Detection: HBV DNA, HCV RNA, and HDV RNA

Molecular markers of viral hepatitis were analyzed in the entire cohort (N = 6773) to detect active infections. HBV DNA was detected in 118 participants (1.7%, 95% CI: 1.4–2.1). A detailed analysis of these cases, including the differentiation between overt and occult HBV infection and their association with demographic and clinical factors, is presented in Section 3.9.

***HCV RNA.*** Among the 127 anti-HCV-positive participants, HCV RNA was detected in 19 cases. This represents 15.0% (95% CI: 9.2–22.4) of seropositive individuals and 0.3% (95% CI: 0.2–0.4) of the overall cohort. HCV RNA positivity was observed exclusively in adults, with no cases detected in children or adolescents. No significant associations were found between HCV RNA positivity and activity group, iatrogenic intervention group, or age.

***HDV RNA.*** Despite 39 participants testing positive for anti-HDV, no HDV RNA was detected in any of these individuals.

### 3.9. Characterization of HBV DNA-Positive Cases: Overt and Occult HB Infection

Of the 118 participants in whom HBV DNA was detected (Section 3.8), only 73 individuals (1.1% of the total cohort, 95% CI: 0.9–1.4) were HBsAg-positive. The remaining 45 participants (0.7% of the total cohort, 95% CI: 0.5–0.9) had undetectable HBsAg, thereby meeting the criteria for occult hepatitis B infection (OBI). OBI was detected across all age groups, including children (Table 8).

Analysis of OBI by professional and social group showed similar prevalence across categories (Table 9). OBI was detected in 0.5% of healthcare workers, 1.2% of educators, 0.9% of preschoolers, and 0.6% of retirees. The frequency of HBsAg-positive cases among pensioners was 1.9%, which was significantly higher than the frequency of OBI (*p* = 0.0089).

A significant association was found between OBI and a history of iatrogenic intervention (surgery or blood transfusion) (Table 10). The prevalence of OBI was 1.4% in individuals with a history of such interventions compared to 0.2% in those without such a history (χ^2^ = 37.7, *p* < 0.0001, OR = 8.54, 95% CI: 3.8–19.4). In contrast, overt HBV infections (HBV DNA+, HBsAg+) were more frequent among individuals without a history of iatrogenic interventions (1.5%) compared to those with such a history (0.4%, χ^2^ = 18.6, *p* < 0.0001).

When stratified by self-reported vaccination and infectious history (as defined in Section 3.7), OBI was detected in 1 of 34 participants in the ‘history-positive and not vaccinated group’ (2.9%, 95% CI: 0.5–14.9), 1 of 16 in the ‘history-positive and vaccinated’ group (6.3%, 95% CI: 1.1–28.3), 10 of 1538 in the naive group (0.7%, 95% CI: 0.3–1.2), and 26 of 3839 in the vaccinated-only group (0.7%, 95% CI: 0.4–1.0). No significant difference in OBI prevalence was observed between the naive and vaccinated-only groups (*p* > 0.05).

Among the 45 OBI cases, the majority (26/45, 57.8%, 95% CI: 42.2–72.3) had no detectable anti-HBc or anti-HBs. Isolated anti-HBs was present in 16/45 (35.6%, 95% CI: 22.0–51.2), isolated anti-HBc in 1/45 (2.2%, 95% CI: 0.1–11.8), and both anti-HBc and anti-HBs were detected in 2/45 (4.4%, 95% CI: 0.5–15.1).

### 3.10. Self-Reported History vs. Laboratory Confirmation

The agreement between self-reported history of hepatitis and the presence of specific markers was analyzed (Table 11, Table 12 and Table 13). No cases of self-reported HD were documented. Self-reported HB was documented in 63 participants (0.9%) and was reported only by individuals aged 30 years and older (Table 11). The frequency of self-reported HB increased with age, from 0.6% in the 30–39 years group to 2.0% in those aged 70+ (Fisher’s *p* = 0.0083).

Among individuals who reported a history of HB, HBsAg, anti-HBc, and HBV DNA were detected significantly more often than in the overall population: 22.2% vs. 1.6% (*p* < 0.0001), 68.3% vs. 11.1% (*p* < 0.0001), and 17.5% vs. 1.7% (*p* < 0.0001), respectively (Table 12). The prevalence of anti-HBs in this group (41.3%) did not differ from the cohort average (*p* > 0.05). Notably, among participants who denied ever having had HB, HBsAg, anti-HBc, and HBV DNA were still detected in 1.4%, 10.6%, and 1.5% of cases, respectively, with prevalence values similar to the cohort average.

Self-reported HC was documented in 29 participants (0.4%), with sporadic cases across all age groups, including one child aged 6–11 years (Table 11). In the group reporting a history of HC, anti-HCV and HCV RNA were detected in 86.2% and 31% of cases. This is significantly higher than the cohort average of 1.8% and 0.3% (*p* < 0.0001), respectively (Table 13). Among those who denied ever having HC, anti-HCV and HCV RNA were found in 1.4% and 0.2% of participants, values consistent with the overall cohort prevalence. Notably, all 39 anti-HDV-positive cases were identified among individuals who reported no history of viral hepatitis.

## 4. Discussion

In this study, a comprehensive assessment of serological and molecular markers of parenteral viral hepatitis B/C/D was conducted for the first time in a representative sample of the conditionally healthy population of St. Petersburg and the Leningrad region (*n* = 6773). Our results show the heterogeneity of the epidemiological conditions in the region. This situation is characterized by: low manifest HB morbidity (HBsAg-positive); a background of significant cumulative exposure to the virus (anti-HBc) in older age cohorts; a moderate burden of HC; and sporadic but widespread prevalence of HD. A key finding of this work is the identification of a significant hidden reservoir of infection represented by OBI (0.7% of the overall cohort). OBI was detected across all age groups, including children.

As a population-based serosurvey of apparently healthy volunteers, our findings reflect the natural distribution of viral markers in the community, which differs fundamentally from clinical cohorts enriched for active disease.

### 4.1. Prevalence of HB Serological Markers and Their Age Dynamics

The seroprevalence patterns we identified reflect both historically established infection risk and the effects of long-term vaccination prophylaxis. The overall HBsAg prevalence (1.7%) is consistent with data from regions with low or intermediate endemicity; it may be a consequence of both vaccination programs and natural population decline [8]. The observed increase in cumulative markers (anti-HBc) with age (from 3.1% in children to 26.4% in those aged ≥70 years, *p* < 0.0001) represents a classic epidemiological pattern. It reflects the accumulation of infection risk over a lifetime in the pre-vaccination era. The sharp increase in the proportion of anti-HBc-positive individuals starting with the 40–49 age group likely marks the cohorts that were at the greatest risk of infection during the 1980s and 1990s, which aligns with global trends for Eastern European countries [10,48]. This epidemiological pattern, characterized by a sharp increase in cumulative HBV markers in cohorts born before the introduction of mass vaccination, is universal and has been described in many countries. Similar age trends, with a peak in HBsAg among middle-aged and older individuals, have been documented in population-based studies in Turkey [49,50], Thailand [51,52], Laos [53], and among refugees in Pakistan [54]. This confirms that the sharp increase we observed in the share of anti-HBc-positive individuals starting with the 40–49 age group reflects a global pattern: a high level of infection in the pre-vaccination era and effective infection control in generations born after immunization programs began [55].

What draws attention is not only the expected increase in the cumulative marker anti-HBc with age, but also the virtually identical strength of the correlation with age for both HBsAg and anti-HBc (r_s_ = 0.88; *p* = 0.0031). This indicates that across most of the age range (from childhood groups to 60–69 years), the prevalence of both markers increases synchronously. This reflects the accumulation of both total infection (anti-HBc) and chronic carriage (HBsAg) among cohorts infected in the pre-vaccination era. This phenomenon may suggest that in cohorts infected with HBV in the pre-vaccination era, HBsAg elimination (either spontaneous or due to therapy) occurred rarely. Consequently, the pool of carriers remained virtually unchanged until reaching the older age groups (60–69 years). However, in the oldest age group (≥70), an expected divergence is observed: the proportion of anti-HBc-positive individuals continues to increase (to 26.4%), while the detection rate of HBsAg decreases (from 2.5% in the 60–69 group to 2.2% in the 70+ group). This dynamic is classically explained by two mechanisms. First, it is due to the natural attrition of some carriers due to mortality, including from HBV-associated diseases (cirrhosis, hepatocellular carcinoma). Second, it may be due to possible spontaneous HBsAg seroconversion in some long-term patients. Thus, the correlation analysis shows an overall trend of marker accumulation with age, whereas sequential analysis of age groups reveals the beginning of elimination of the carrier pool in the oldest cohorts.

In contrast, the maximum anti-HBs detection rate in children (69.9% in the 1–5 years group), and its subsequent progressive decline with age (r_s_ = −0.8, *p* = 0.0138), directly reflects the success of routine childhood vaccination, which began in Russia in the late 1990s. The decline in Ab levels over time following vaccination is a well-known phenomenon, underscoring the importance of monitoring population immunity to determine the need for revaccination [56,57]. The higher share of seroprotected individuals among young adults (18–29 years, 59.9%) compared to adolescents may be explained by both later vaccination (e.g., in higher education or upon employment) and by a boosting effect from contact with the virus.

A detailed analysis of anti-HBs levels revealed two age-related peaks of high Ab concentration (≥150 mIU/mL): one in the age group 1–5 years (32.8%) and another among young adults aged 18–29 years (29.5%). The first peak is unequivocally associated with the post-vaccination immune response within the national immunization schedule. The second may reflect both a boosting effect from natural contact with the virus in seroconverted individuals and/or later vaccination, such as upon enrollment in higher education or upon biomedical employment. If the rise in anti-HBs in young adults were due to natural infection (sexual or other contact with HBV), it should be accompanied by a corresponding increase in anti-HBc. To test this, we examined the distribution of serological profiles according to self-reported vaccination and hepatitis history (Appendix A, Table A10). The “anti-HBs only” profile was observed in 45.1% of vaccinated individuals with no history of hepatitis (HNV group, *n* = 3839), compared to only 16.3% of unvaccinated individuals with no history of hepatitis (HNNV group, *n* = 1538). Conversely, isolated anti-HBc was rare in the HNV group (1.5%) but more common in those with a history of infection (17.6% in HPNV). These data confirm that the peak in anti-HBs seroprevalence among young adults primarily reflects vaccine-induced immunity (including catch-up vaccination) rather than natural exposure, although a boosting effect from subclinical infection in vaccinated individuals cannot be completely excluded. A sharp increase in the share of seronegative individuals during adolescence (from 30.1% in the 1–5 years group to 65.7% in the 12–17 years group) is noteworthy. This reflects a predictable decline in post-vaccination Abs 10–15 years after immunization and raises the question of the feasibility of revaccination in this age group [56,57]. The highest proportion of seronegative individuals found among those aged 60–69 years and 70+ years (70.2% and 66.2%, respectively) is due to both low vaccination coverage in these subgroups and a natural decline in Ab levels over time among previously vaccinated or infected individuals.

Our data on the age dynamics of anti-HBs (a decline in seroprotection from 69.9% in children aged 1–5 years to 29.8% in individuals aged 60–69 years) are consistent with the results of federal serological monitoring. According to that monitoring, the share of protected individuals among children aged 3–4 years in Russia was 65.8–72.5%, while among adults it did not exceed 70% [58]. The authors of that study also noted that the strength of post-vaccination immunity in adolescents aged 16–17 years (49.6–64.9%) is insufficient, and it raises the question of the feasibility of booster immunization in the subgroup. Our data on the sharp increase in the share of seronegative individuals during adolescence (from 30.1% in the 1–5 years group to 65.7% in the 12–17 years group) fully confirm this conclusion and justify the need to discuss measures to maintain population immunity.

### 4.2. Distribution of Hepatitis B Serological Profiles

Analysis of HB serological profiles enables reconstruction of the structure of population immunity and identification of groups at potential risk of reactivation or occult carriage. The most common profile in the studied population was “anti-HBs only” (34.3%). Although this pattern primarily indicates post-vaccination immunity, it can also be observed in individuals who have resolved an infection with subsequent loss of anti-HBc after decades [6,30]. The inability to distinguish between these two states underscores the importance of documented vaccination records in epidemiological studies. The profile indicating resolved infection (anti-HBc+ anti-HBs+) was identified in 8.3% of participants. This group generally does not require follow-up, but should be informed of their status to avoid unnecessary revaccination.

Individuals with “anti-HBc only” (2.5%) warrant particular attention. This serological pattern can reflect two distinct situations: infection in the distant past with loss of anti-HBs, or the presence of OBI [28,29]. This justifies the need for HBV DNA testing in this category of individuals, especially when planning immunosuppressive therapy.

Rare profiles, including the simultaneous presence of HBsAg and anti-HBs (0.4%), may indicate infection with different viral subtypes or ongoing seroconversion. This atypical serological profile may be associated with viral immune escape due to mutations in the S gene, particularly within the “a” determinant, which reduces Ab recognition of HBsAg [59,60,61,62]. Additionally, some individuals may have heterologous subtype-specific Abs that do not correspond to the circulating viral subtype [63]. Several studies have linked this profile to progressive liver disease, especially hepatocellular carcinoma [59,64,65]. This underscores the need for dynamic follow-up of such patients, although some cohorts have shown no clear association with worse outcomes [59,64,65,66,67].

Finally, more than half of the individuals analyzed (53.3%) had no HB markers. This group includes both truly naïve individuals and those who have been vaccinated, yet whose anti-HBs levels have fallen below the protective level. It is important to emphasize that seronegative status does not guarantee the absence of OBI. As such, molecular screening is justified in at-risk groups.

### 4.3. Comparative Analysis of HB Vaccinated and Unvaccinated Groups

The data from our study convincingly show the effectiveness of vaccination prophylaxis. Among vaccinated individuals, the share infected (anti-HBc: 8.7% vs. 15.4% in unvaccinated individuals) and HBsAg carriers (1.3% vs. 2.2%, OR = 1.7) were significantly lower. This confirms that vaccination not only protects against clinically manifest infection, but also reduces the overall risk of infection [39]. However, the detection of HBV markers in vaccinated individuals requires explanation. We propose three main, non-mutually exclusive explanations for this phenomenon.

First, the most likely cause is HBV infection that occurred before vaccination. In Russia, as in many other countries, mandatory pre-vaccination testing for HBV markers (HBsAg, anti-HBc) is not performed. Vaccination begins in the neonatal period, and if the mother is an HBsAg carrier, the risk of perinatal viral transmission remains extremely high (up to 95%), despite the timely administration of the first vaccine dose and immunoglobulin [8,9]. Additionally, individuals may have become infected during later childhood or adulthood before vaccination, for example, within immunization programs for certain occupational groups (healthcare workers, students).

Second, the possibility of infection with viral variants carrying escape mutations in the S gene, which encodes HBsAg, cannot be excluded. Such mutations can alter the antigenic structure of the surface protein, making it less recognizable to vaccine-induced Abs [18,38]. As a result, vaccine-induced immunity may be insufficient to completely eliminate such a mutant virus, leading to the development of breakthrough infection. Third, in some cases, viral contact may indeed have occurred after post-vaccination immunity was already established. In this scenario, the infection may be abortive or transient, leaving behind anti-HBc, often without detectable HBsAg [6,30].

Analysis of anti-HBs levels showed that, vaccinated individuals not only had a higher frequency of seroprotection (52.2% vs. 26.9%), but also a qualitatively better immune response (23.8% had high titers ≥150 mIU/mL vs. 12.3% in the unvaccinated). Our data on higher seroprotection rates and Ab levels in vaccinated individuals are consistent with global practice [68,69]. Our findings regarding the decline in the share of seroprotected individuals with age align with the results of a major meta-analysis assessing the duration of post-vaccination immunity. According to that study, 10 years after childhood vaccination, protective anti-HBs levels are maintained in only 63.2% of vaccinated individuals, with an annual decline of 6.62% [70]. It is important to emphasize that the duration of maintaining a protective Ab level directly depends on the initial titer after completing vaccination. A 30-year prospective study of healthcare workers showed that: in individuals with an initial titer ≥1000 mIU/mL, Abs persisted for an average of 31.0 years; with levels of 100–999 mIU/mL, they persisted for 25.4 years; and with a low titer (10–99 mIU/mL), they lasted only 19.2 years [71].

The aforementioned findings explain the presence of seronegative individuals even among the vaccinated, and justify the rationale for booster immunization in those with an initially low response. We acknowledge that waning anti-HBs levels do not necessarily indicate loss of immune memory, as cellular immunity may persist. The WHO currently does not recommend universal booster vaccination for populations with a completed primary series. However, our data on the high proportion of seronegative adolescents and young adults, combined with the documented risk of OBI, support the rationale for targeted booster strategies in specific risk groups, while noting that this remains an area of ongoing debate. In addition to natural Ab decline over time, the absence of protective immunity in some vaccinated individuals may be associated with an initial non- or hypo-response to vaccination. The frequency of such non-response is 5–10% in the population [72]. Recent evidence from a 24-year cohort study by Fonzo et al. show that modifiable behavioral factors, such as smoking, significantly increase the risk of losing protective Abs [73]. This is particularly relevant in the context of our aging population.

Smoking exposure is cumulative. Older individuals have had more years of potential exposure, and historical data indicate that smoking prevalence among older generations in Russia has been substantially higher than among younger cohorts [74]. Among factors associated with inadequate Ab levels (age, smoking), vitamin D deficiency stands out insofar as it increases the odds of lacking a protective titer by more than 2.5-fold. This is particularly relevant to the St. Petersburg area, which is located in a low-insolation zone. According to a population-based study, approximately 90% of the city’s residents have insufficient vitamin D levels [75].

It is important to note that interpretation of the “anti-HBs only” profile is not always straightforward. Studies show that, with this profile, persistence of HBV DNA with a low viral load, meeting the criteria for OBI, is possible, particularly in cohorts of young vaccinated adults [66,76,77,78,79,80]. In such cases, the risk of infection reactivation under immunosuppression remains, which requires attention when managing these patients [68,69].

Interestingly, even in the “naïve” group (those denying illness or vaccination), 27% had anti-HBs Abs. This may be explained either by forgotten vaccination (especially in older adults) or by a past asymptomatic infection that led to Ab production, but left no trace in the form of anti-HBc (the so-called “anti-HBs only” post-infectious profile). Correlation analysis confirmed a strong inverse relationship between age and vaccination coverage (r_s_ = −0.95; *p* = 0.0004), with age accounting for more than 90% of the variance in vaccination rates (R^2^ = 0.904). The extremely low vaccination rate among individuals over 70 years of age (39%), combined with their highest infection rate (anti-HBc 26.4%), confirms that this group is at the greatest risk of severe outcomes in the event of accidental infection or reactivation.

The data obtained justify the need not only to maintain high vaccination coverage among newborns, but also to implement catch-up and targeted immunization programs for vulnerable adult populations. According to the current National Immunization Schedule, HB vaccination is strongly recommended for individuals at risk, including healthcare workers, family members of HBsAg carriers, and patients receiving blood transfusions or undergoing hemodialysis [39]. The decline in anti-HBs levels we observed in adolescents and young adults also highlights a potential need for revaccination in these cohorts to maintain long-term protection [56,57].

### 4.4. Iatrogenic Interventions and HBV Infection: Manifest and Occult Forms

Analysis of the association between a history of iatrogenic interventions (surgery, blood transfusion) and markers of parenteral hepatitis revealed fundamentally different patterns for different forms of HBV infection and for other viruses. The most significant finding was the detection of a strong association between iatrogenic interventions and OBI. The likelihood of detecting OBI in individuals who had undergone surgery or blood transfusion was more than eight-fold higher than in those without such interventions (1.4% vs. 0.2%, OR = 8.54). The strong association we found between OBI and iatrogenic interventions is consistent with data showing that the risk of transmission via percutaneous injuries is 6–30% for HBV [81]. Medical procedures, particularly in the era before strict screening and sterilization standards were introduced, created conditions for infection with the subsequent formation of occult carriage. This is also supported by analyses of outbreaks in healthcare settings [82]. The extremely low infectious dose of HBV (as few as 16 copies) and its high environmental stability made transmission possible in healthcare facilities in past decades even with minimal breaches of skin or mucous membrane integrity [37].

In contrast, manifest forms of HBV infection (HBsAg-positive) were significantly more common among individuals who denied iatrogenic interventions (1.5% vs. 0.4%, *p* < 0.0001). This apparent paradox may have several explanations. First, individuals with manifest infection identified in our study likely represent a cohort infected primarily during early childhood (perinatally or horizontally within the family), when the risk of chronicity is highest, reaching up to 95% [8,9]. Such infection, established at an early age, naturally presents as classical HBsAg-positive carriage. Second, it cannot be ruled out that some individuals who underwent iatrogenic interventions may have been vaccinated later in life (e.g., upon employment in healthcare). Vaccination could have prevented the development of a manifest form, but may not have protected against the establishment of OBI following subsequent contact with the virus. Third, iatrogenic interventions may have occurred after the patient had already developed an immune response to natural infection (for example, in older adults who had the infection in their youth). In this case, these interventions did not create a “new” risk of manifest infection.

Thus, iatrogenic interventions were strongly associated with OBI, suggesting a possible contribution to the formation of this hidden reservoir, whereas manifest carriage (HBsAg+) was primarily associated with other, non-iatrogenic transmission routes. This underscores the need to include highly sensitive molecular methods in the screening of individuals with a history of iatrogenic procedures to identify hidden sources of infection.

### 4.5. Occult Hepatitis B Infection (OBI) as a Hidden Reservoir of Infection

The most significant finding of our work is the identification of a high frequency of OBI (0.7%). Official morbidity data are based on HBsAg detection. As such, it is reasonable to assume that official statistics would be significantly higher if the contribution of OBI were included. This has both clinical and epidemiological significance. The OBI frequency we identified (0.7% of the overall cohort) is almost identical to the estimated global prevalence of OBI, which, according to a 2022 meta-analysis, is 0.82% [41]. This similarity indicates that, despite regional characteristics, the population we studied aligns with the global pattern of OBI prevalence. It is important to emphasize that assessment of the OBI burden can be approached from different perspectives.

In our study, we estimated the share of individuals with occult infection in the entire population. Another important measure is the share of OBI among all individuals with detectable HBV DNA (i.e., among the total pool of infected individuals). This proportion reflects the structure of the infectious reservoir. For example, a study among blood donors in Dagestan showed that occult forms accounted for only 6.7% of infected individuals [83]. In contrast, our study revealed a fundamentally different pattern. Among all participants with detectable HBV DNA (*n* = 118), only 73 (61.9%) were HBsAg-positive, while the remaining 45 (38.1%) were cases of OBI. Thus, in our population, more than one-third of all ongoing infections occur in an occult, HBsAg-negative form. This shows that despite a comparable overall prevalence of OBI in the population, its contribution to the structure of the infectious reservoir can be extremely high. Comparison of our OBI serological profiles with other studies reveals both similarities and important differences. In a Croatian study focusing on “anti-HBc only” individuals, OBI was detected in 10.6% of such cases [84]. A Greek blood donor study also restricted OBI detection to anti-HBc-positive individuals [85]. In contrast, an Asian study reported that 11% of OBI cases showed an “anti-HBs only” profile without evidence of vaccination [86], which aligns with our finding of 35.6% isolated anti-HBs among OBI cases. Notably, 57.8% of OBI cases in our study had no detectable anti-HBc or anti-HBs, a finding rarely reported elsewhere because most studies limit OBI screening to anti-HBc-positive individuals. A recent meta-analysis by Jiaying Wu et al. (2024), which included 50 studies (12,977 participants) and identified 21 studies (5775 participants) with HBsAg-negative/anti-HBc-negative status, reported an OBI prevalence of 3.0% in this seronegative population [87]. In our study, the overall OBI prevalence was 0.7% (45/6773), while the prevalence of seronegative OBI was 0.4% (26/6773). These figures are consistent with the 0.8% prevalence reported for healthy populations in the same meta-analysis. These data confirm that serological markers alone cannot reliably identify occult carriers and that molecular testing is required.

The detection of OBI in all age groups, including children (1.0% in the 1–17 years group), suggests that establishment of the occult reservoir may occur from an early age. This may be related to perinatal transmission, which is known to lead to chronicity in 95% of cases [8], or to infection in early childhood with subsequent HBsAg seroconversion. A meta-analysis examining the prevalence of OBI in individuals under 18 years of age showed that it ranges from 2.1% in low-risk groups to 9.7% in high-risk groups [88]. Our data (1.0% in the 1–17 years group) are at the lower bound of these estimates. This may indirectly reflect the effectiveness of perinatal and early horizontal HBV transmission prevention programs in the region.

As discussed in detail above (Section 4.4), iatrogenic interventions proved to be the strongest predictor of OBI (OR = 8.54), underscoring the role of medical procedures from past decades in the formation of the occult reservoir. Beyond healthcare facilities, the high environmental stability and extremely low infectious dose of HBV enable household transmission through shared hygiene items [37].

The clinical significance of OBI is well established. These individuals are at risk of viral reactivation under immunosuppression (e.g., during chemotherapy or organ transplantation) [31,32]. They also have a potentially higher risk of developing HCC, particularly in the context of other liver diseases [33,34,35].

Analysis of OBI distribution across different occupational and social groups revealed heterogeneity in the structure of the occult reservoir. Notably, among retirees, the frequency of manifest HBsAg-positive forms (1.9%) was significantly higher than the frequency of OBI (0.6%, *p* = 0.0089). This aligns with the hypothesis that, in older age cohorts, infected primarily in the pre-vaccination era, chronic HBV infection is more likely to present in the classical HBsAg-positive form. In contrast, in younger groups, occult forms may constitute a substantial proportion of the infectious reservoir. For example, in the preschool and schoolchild groups, the ratios of “HBsAg-positive cases to OBI” were approximately 1:4 and 1:7, respectively. This observation underscores that epidemiological surveillance focused solely on HBsAg detection may underestimate the burden of infection precisely in younger age groups, where OBI plays a relatively larger role.

It is important to note that we found no statistically significant difference in OBI frequency between vaccinated and unvaccinated individuals (0.7% in both groups) who denied a history of HBV infection. Although hepatitis B vaccines are highly effective in preventing clinical disease and chronic HBsAg carriage, they do not necessarily confer absolute sterilizing immunity. Transient or occult HBV infection may rarely occur after exposure, but effective immune control prevents persistent replication or chronic infection.

Interestingly, the structure of HBV infection in the general population we studied differed from that observed among pregnant women in the same region [89]. In our study, for every case of manifest infection (HBsAg+/DNA+), there were 0.61 cases of OBI. It is important to note that the proportion of HBV DNA detection among HBsAg-positive individuals varies substantially depending on the study population and assay characteristics. In population-based serosurveys, this proportion is often lower than in clinical cohorts due to the higher proportion of individuals with low-level or intermittent viremia, which is characteristic of population-based cohorts. For instance, studies in tribal populations in India and hospital patients in Somalia reported HBV DNA detection in 42.9% and 19.4% of HBsAg-positive individuals, respectively, despite using assays with comparable or lower sensitivity [90,91]. Furthermore, individuals with low-level HBsAg expression are known to have significantly lower rates of HBV DNA detectability [92,93]. Our finding of 63% HBV DNA positivity among HBsAg-positive participants is therefore consistent with the expected distribution in a community-based sample and does not indicate an assay limitation. However, among pregnant women, OBI predominated over manifest forms at a ratio of 1.46:1. The overall detection rate of HBV DNA in pregnant women was also substantially higher (4.68% vs. 1.74% in our sample), despite comparable HBsAg positivity rates (1.90% and 1.70%, respectively). Such pronounced differences in the frequency of HBV DNA detection and the structure of infection may be attributed to several factors.

First and foremost, pregnant women represent a group with inherently high sexual activity. Given the fact of pregnancy, a high likelihood of unprotected activity exists, currently one of the main transmission routes for HBV [94,95]. Our study also included all population groups, including children, adolescents, and older adults. Understandably, this lowers the average frequency of infectious markers. Furthermore, multiple pregnancies are associated with higher detection rates of anti-HBc, HBsAg, and HBV DNA in pregnant women [89]. They also involve a greater number of medical interventions, which also constitute a risk factor. Thus, pregnant women may be considered a sentinel group that accumulates the main risks of HBV infection in the population of reproductive age, thereby explaining the higher detection rates of both manifest and occult forms of infection.

It is important to note that the detection of OBI may be associated not only with characteristics of the host immune status, but also with the molecular genetic characteristics of the virus itself. In the aforementioned study of pregnant women in St. Petersburg, it was shown that HBsAg-negative forms of HBV are associated with the circulation of viral variants carrying escape mutations in the major hydrophilic region (MHR) of the S gene, including G145R, P120T, Q129R, and others [89]. These mutations alter the antigenic structure of HBsAg, allowing the virus to evade both detection by diagnostic test systems and neutralization by vaccine-induced Abs [96,97,98]. Recent findings from Li et al. further show that HBV quasispecies characteristics and specific mutations in the PreC region are associated with OBI development in infants born to highly viremic mothers, even after immunoprophylaxis. This highlights the role of viral genetic factors in establishing occult infection early in life [99]. In our cohort, 45 cases of OBI were identified, and their molecular genetic characterization will be the subject of further research. Such work is necessary to assess the potential risks associated with the circulation of escape mutants in the population, including possible impacts on the effectiveness of vaccination prophylaxis and diagnostics.

### 4.6. Hepatitis C and D: Epidemiology and Diagnosis

The prevalence of anti-HCV (1.9%) in our study falls within estimates for the general population of Russia and Europe [7,11]. The absence of a clear age trend and the presence of anti-HCV even in younger age groups (1.2% in children aged 1–17 years) indicates that, despite the dominance of the parenteral transmission route among young individuals in the past, the virus continues to circulate today, possibly due to other transmission routes or diagnostic inaccuracies. The detection of HCV RNA in only 15% of anti-HCV-positive individuals (0.3% of the overall cohort) may reflect a high rate of spontaneous viral elimination (approximately 20–30%) and/or the effectiveness of antiviral therapy. The latter has led to the cure of a significant proportion of infected individuals [22]. These figures align with recent population-based screening programs in Europe, where only 12.5–28.3% of anti-HCV-positive individuals had detectable HCV RNA, reflecting the impact of spontaneous clearance and widespread DAA therapy in the general population [100,101]. Nevertheless, the presence of RNA-positive cases, particularly among individuals denying infection, points to a persistent problem of hidden sources of infection. This underscores the need to transition from screening only for Abs to broader use of molecular methods for detecting active infection. A recent large-scale serosurvey across 12 Russian regions (*n* = 42,000) reported anti-HCV prevalence ranging from 1.1 to 1.4% in Moscow, and Belgorod to 1.8–2.1% in Dagestan, Tatarstan, Novosibirsk, Tyva, and southern Yakutia, and up to 3.4–5.2% in Khabarovsk Krai and Arctic Yakutia [102]. Our anti-HCV estimate of 1.9% in Northwest Russia is consistent with the moderate prevalence group identified in that study.

False-negative serology cannot be entirely excluded, particularly in the early window period or in immunocompromised individuals. However, our study population consisted of apparently healthy volunteers, and the consistency of our estimates with national data suggests that the impact of false-negative results is likely minimal.

The detection rate of anti-HDV was 0.6%. Comparison with other Russian regions shows substantial heterogeneity. In the Republic of Tyva, a highly endemic region, anti-HDV prevalence among HBsAg-positive individuals reached 32.7% in clinical registry data and 1.0% in the general population [103]. In the Arctic zone of Yakutia, anti-HDV was detected in 29.4% of HBsAg-positive indigenous adults [104]. By contrast, in Moscow, HDV prevalence among HBsAg-positive children was 4.7% [105], consistent with the description of the European part of Russia as a region with low HDV infection rates. However, population-based HDV prevalence studies in Russia remain scarce outside highly endemic regions and risk groups. Therefore, our finding of 0.6% anti-HDV seroprevalence in the general population of Northwest Russia (with no HDV RNA detected among anti-HDV-positive individuals) provides important baseline data for a region where such estimates have been lacking.

The absence of HDV RNA in all 39 anti-HDV-positive patients is an important and unexpected finding. It may have several explanations. First, it could be a consequence of spontaneous or treatment-induced clearance of HDV with persisting Abs. Second, effective suppression of HDV may occur in the context of low HBV replication. A third possibility is a very low level of viremia in patients, below the detection threshold of the method (100 IU/mL). We also note that no commercial confirmatory assay was available for anti-HDV, so false-positive serology cannot be entirely excluded. This raises the question of the need to use more sensitive methods for detecting HDV RNA, especially when occult or low-level infection is suspected. The absence of detectable HDV RNA in all anti-HDV-positive individuals in our study, despite 0.6% seroprevalence in the general population, contrasts with reports from neighboring Romania. There, active HDV replication was detected in 75.6% of anti-HDV-positive cases among HBsAg carriers [106]. While direct comparison is limited by different study populations, these contrasting findings highlight significant regional heterogeneity in HDV epidemiology across Eastern Europe and underscore the importance of systematic screening in HBsAg-positive cohorts.

Analysis of the association of these infections with iatrogenic interventions did not reveal significant associations, although there was a trend toward a higher frequency of anti-HCV and anti-HDV among individuals who had undergone surgery or blood transfusion. The lack of significance may be related to the relatively small number of observations for these markers, as well as to the possibility that the main transmission routes for HCV and HDV in the studied population may have been different (intravenous drug use, sexual transmission). Furthermore, it cannot be ruled out that the majority of iatrogenic infections with these viruses occurred in earlier historical periods and are no longer reflected in current seroprevalence due to mortality or viral elimination [22]. This may also reflect a shift in the dominant transmission routes for these infections in recent decades, despite their well-documented roles in the past [107,108].

### 4.7. Analysis of Agreement Between Self-Report and Laboratory Data

A key step in epidemiological analysis is assessing the structure of the infectious status of the population with respect to parenteral viral hepatitis based on comparing participant self-reports (including confirmed ones) with verified laboratory data. Such comparison enables evaluation of the population’s awareness of their infectious status, identification of cases of asymptomatic or subclinically resolved infection, and analysis of the reliability of the anamnestic method as a screening tool [23,24,26]. Here, we conducted a comprehensive assessment of the agreement between volunteer subjective responses and objective data on the presence of specific serological markers for all three infections.

***Hepatitis B.*** Among individuals who reported a history of HB, HBsAg and anti-HBc were detected significantly more often than in the general population (22.2% vs. 1.6% and 68.3% vs. 11.1%, respectively), confirming the reliability of self-report for manifest forms. Among those who denied ever having had HB, HBsAg was detected in 1.4% of cases and anti-HBc in 10.6%. These 1.4% of HBsAg-positive individuals unaware of their status represent an active but hidden source of infection, invisible to official statistics. An even larger proportion (10.6%) of individuals with anti-HBc who denied infection indicates the widespread prevalence of asymptomatically resolved HB, which was never diagnosed and left no subjective memory of the illness.

***Hepatitis C.*** Among respondents who reported a history of HC, anti-HCV was confirmed in 86.2% of cases, reflecting the high specificity of self-report. However, in the group that denied having HC, anti-HCV was detected in 1.4% of participants, which is comparable to the general cohort prevalence (1.8%). This means that a significant proportion of anti-HCV carriers (and potentially those with active infection) are unaware of their status. The detection of HCV RNA in 31% of individuals aware of their diagnosis, compared to 0.2% among those who denied a history of the disease, underscores that it is informed patients who are more likely to be under medical follow-up and receiving therapy, while undiagnosed cases remain outside the healthcare system [24,25].

***Hepatitis D.*** All 39 anti-HDV-positive cases were identified among individuals who denied a history of the disease. This is entirely consistent with the understanding of HDV as a “satellite” of HBV, which often remains undiagnosed due to the lack of routine screening and low awareness among both patients and physicians [11,12]. This fact underscores the critical importance of including anti-HDV testing in the diagnostic algorithm for all HBsAg-positive individuals.

It is important to note that among individuals who reported a history of HB, 31.7% had neither HBsAg nor anti-HBc detected. This may reflect either cases of mistaken self-identification (e.g., confusion with other liver diseases) or loss of serological markers decades after infection. Similarly, 13.8% of respondents who reported a history of HC tested negative for anti-HCV. This may be a consequence of a misdiagnosis made in the past based solely on clinical data without serological confirmation. These findings are consistent with global estimates indicating that up to 80–90% of individuals infected with HBV, and up to 50% of those infected with HCV, may be unaware of their status, particularly in populations with low health literacy and limited access to diagnostics [23,24,26]. The observed discrepancy underscores the critical importance of shifting from passive detection (based on patient presentation) to active screening, especially among at-risk groups and older age cohorts, where the proportion of undiagnosed cases is highest. Only a comprehensive approach combining serological and molecular testing can provide a reliable assessment of the true burden of infection and bring us closer to the elimination goals set by the WHO for 2030 [109].

### 4.8. Limitations of the Study

When interpreting the results, several limitations must be considered. First, the cross-sectional design of the study allows for the assessment of marker prevalence and associations between variables at the time of sample collection, but does not permit establishment of causal relationships or the tracking of serological and molecular markers over time. For example, we cannot confidently determine whether vaccination preceded infection or vice versa in individuals with detectable anti-HBc and anti-HBs. Second, although data on HB vaccination and history of disease were verified using medical records (vaccination certificates, medical charts), information on some other epidemiologically significant factors (parenteral interventions, absence of a history of disease) was obtained through participant self-report. While we made every effort to verify this information using medical records where available, recall bias remains a potential limitation, particularly for older adults reporting events that occurred decades ago. Third, our cohort had a substantial overrepresentation of females (73.6%), reflecting volunteer bias common in population-based serosurveys. This may limit the generalizability of sex-specific estimates for markers of parenteral hepatitis. However, previous studies have not shown major sex differences in HBV seroprevalence after adjusting for age and risk factors, and for HCV and HDV, sex-specific population data in Russia are limited. Fourth, the use of qualitative PCR analysis with a sensitivity threshold of 100 IU/mL for HCV and HDV may have resulted in the under-detection of cases with extremely low viremia; this is particularly relevant for latent forms of infection. However, this threshold is consistent with standard practice in population-based surveys and is sufficient for estimating prevalence at the community level, as demonstrated by concordance with regional and global meta-analyses. For anti-HDV, no confirmatory assay was available, so the possibility of false-positive results cannot be entirely excluded. Fifth, the absence of data on HBV, HCV, and HDV genotypes limits understanding of molecular epidemiology and the circulation of potential escape mutants in the context of OBI. Finally, this study was conducted in St. Petersburg and the Leningrad region. While our findings provide valuable insights into the epidemiology of viral hepatitis in Northwest Russia, extrapolation to other regions with different epidemiological profiles, vaccination policies, and treatment coverage should be made with consideration of these local characteristics.

## 5. Conclusions

This comprehensive serological and molecular study in Northwest Russia is the first to combine population-level serology and molecular detection of HBV, HCV, and HDV, including OBI, in this region, and reveals a substantial hidden reservoir of HBV infection. OBI was found to constitute a significant proportion of all active HBV infections, including a strong association with a history of iatrogenic interventions. The presence of OBI across all age groups, including children, confirms that HBsAg screening alone substantially underestimates the true HBV burden. This underscores the need for integrating nucleic acid testing into screening algorithms for risk groups. The low detection rate of HCV RNA among anti-HCV-positive individuals and the absence of HDV RNA in all anti-HDV-positive subjects, suggest effective viral clearance or low-level replication. Nonetheless, the presence of anti-HCV and anti-HDV in the population warrants continued surveillance. High rates of unrecognized infection and waning vaccine-induced immunity highlight critical gaps in public awareness and current immunization strategies.

These findings provide an evidence-based rationale for optimizing diagnostic algorithms, strengthening surveillance, and considering booster vaccination strategies to achieve viral hepatitis elimination goals. Future studies should focus on molecular genetic characterization of the detected OBI isolates, including comparative analysis with HBV strains previously identified in regional high-risk groups. This will be essential for elucidating transmission patterns, assessing the potential prevalence of clinically relevant vaccine escape mutants, and ultimately refining vaccination strategies.

## Figures and Tables

**Table 1:** Serological marker prevalence by age group.

Age Group, Years	N	HBsAg			Anti-HBc			Anti-HBs			Anti-HCV			Anti-HDV		
		*n*	%	95% CI	*n*	%	95% CI	*n*	%	95% CI	*n*	%	95% CI	*n*	%	95% CI
1–17	1415	3	0.2 #	0.0–0.6	44	3.1	2.3–4.1 #	698	49.3 *	46.7–52	17	1.2	0.8–1.9	5	0.4	0.2–0.8
1–5	369	2	0.5	0.1–1.9	8	2.2	1.1–4.2 #	258	69.9 *	65.1–74.4	5	1.4	0.6–3.1	1	0.3	0.0–1.5
6–11	510	0	0	0.0–0.7	18	3.5	2.2–5.5 #	256	50.2 *	45.9–54.5	9	1.8	0.9–3.3	2	0.4	0.1–1.4
12–17	536	1	0.2 #	0.0–1.0	18	3.4	2.1–5.2 #	184	34.3 #	30.4–38.4	3	0.6	0.2–1.6	2	0.4	0.1–1.4
18–29	796	10	1.3	0.6–2.3	17	2.1	1.3–3.4 #	477	59.9 *	56.5–63.3	8	1	0.5–2.0	4	0.5	0.2–1.3
30–39	838	16	1.9	1.1–3.1	42	5	3.7–6.7 #	464	55.4 *	52.0–58.7	10	1.2	0.6–2.2	4	0.5	0.2–1.2
40–49	914	20	2.2	1.3–3.4	108	11.8	9.9–14.1	363	39.7	36.6–42.9	23	2.5	1.7–3.7	3	0.3	0.1–1.0
50–59	900	22	2.4	1.5–3.7	125	13.9	11.8–16.3	305	33.9 #	30.9–37.0	22	2.4	1.6–3.7	8	0.9	0.5–1.7
60–69	930	23	2.5	1.6–3.7	173	18.6	16.2–21.2 *	277	29.8 #	26.9–32.8	20	2.2	1.4–3.3	6	0.6	0.3–1.4
70+	980	22	2.2	1.4–3.4	259	26.4	23.8–29.3 *	331	33.8 #	30.9–36.8	27	2.8	1.9–4.0	9	0.9	0.5–1.7
**Total**	**6773**	**116**	**1.7**	**1.4–2.1**	**768**	**11.3**	**10.6–12.1**	**2915**	**43**	**41.9–44.2**	**127**	**1.9**	**1.6–2.2**	**39**	**0.6**	**0.4–0.8**

Note: * significantly higher than the total value; # significantly lower than the total value; *p* < 0.05 for all comparisons.

**Table 2:** Distribution of HB serological markers in the study group (N = 6773).

Hepatitis B Markers	*n*	%	95% CI
HBsAg only	51	0.8	0.6–1.0
Anti-HBs only	2320	34.3	33.1–35.4
Anti-HBc only	167	2.5	2.1–2.9
HBsAg, anti-HBs	26	0.4	0.3–0.6
HBsAg, anti-HBc	30	0.4	0.3–0.6
HBsAg, anti-HBs, anti-HBc	9	0.1	0.1–0.3
Anti-HBs, anti-HBc	560	8.3	7.6–8.9
Any of the following: HBsAg, anti-HBs, anti-HBc	3163	46.7	45.5–47.9
The aforementioned markers are absent	3610	53.3	52.1–54.5

**Table 3:** Hepatitis B/C/D markers by intervention history (surgery, blood transfusion).

History of Surgery or Blood Transfusion	N	HBsAg			Anti-HBc			Anti-HBs			Anti-HCV			Anti-HDV		
		*n*	%	95% CI	*n*	%	95% CI	*n*	%	95% CI	*n*	%	95% CI	*n*	%	95% CI
Yes	2632	31	1.2	0.8–1.7	364	13.8 *	12.6–15.2	1054	40	38.2–41.9	60	2.3	1.8–2.9	17	0.6	0.4–1.0
No	4141	85	2.1	1.6–2.5	404	9.8	8.9–10.7	1861	44.9	43.4–46.5	67	1.6	1.3–2.0	22	0.5	0.4–0.8
Total	6773	116	1.7	1.4–2.1	768	11.3	10.6–12.1	2915	43	41.9–44.2	127	1.9	1.6–2.2	39	0.6	0.4–0.8

Note: * significantly higher than the total value; *p* < 0.05 for all comparisons.

**Table 4:** Hepatitis B vaccination status by age group ^†^.

Age Group, Years	N	Vaccinated Against HBV			Not Vaccinated Against HBV		
		*n*	%	95% CI	*n*	%	95% CI
1–17	1330	1209	90.9 *	89.2–92.3	121	9.1 #	7.7–10.8
1–5	354	323	91.2 *	87.8–93.8	31	8.8 #	6.2–12.2
6–11	479	428	89.4 *	86.3–91.8	51	10.6 #	8.2–13.7
12–17	497	458	92.2 *	89.5–94.2	39	7.8 #	5.8–10.5
18–29	670	585	87.3 *	84.6–89.6	85	12.7 #	10.4–15.4
30–39	670	526	78.5 *	75.2–81.5	144	21.5 #	18.5–24.8
40–49	721	488	67.7	64.2–71.0	233	32.3	29.0–35.8
50–59	702	448	63.8 #	60.2–67.3	254	36.2 *	32.7–39.8
60–69	725	369	50.9 #	47.3–54.5	356	49.1 *	45.5–52.7
70+	679	265	39 #	35.4–42.7	414	61 *	57.3–64.6
Total	5497	3890	70.8	69.5–72.0	1607	29.2	28.0–30.5

Note: * significantly higher than the total value; # significantly lower than the total value; *p* < 0.05 for all comparisons. ^†^ Mandatory universal HBV vaccination for newborns was introduced in Russia in 1998. The current schedule (0–1–6 months) was fully implemented by 2001. First dose: within 24 h of birth; second: at 1 month; third: at 6 months. High-risk infants (born to HBsAg-positive mothers) receive a fourth dose at 12 months (0–1–2–12 schedule).

**Table 5:** Hepatitis B and D viral markers by vaccination status.

Status	N	HBsAg			Anti-HBc			Anti-HBs			Anti-HDV		
		*n*	%	95% CI	*n*	%	95% CI	*n*	%	95% CI	*n*	%	95% CI
Vaccinated against HBV	3890	50	1.3	1.0–1.7	340	8.7 #	7.9–9.7	2031	52.2 *	50.6–53.8	22	0.6	0.4–0.9
Not vaccinated against HBV	1607	35	2.2	1.5–3.0	248	15.4 *	13.7–17.3	433	26.9 #	24.8–29.2	11	0.7	0.4–1.2
Total	5497	85	1.5	1.2–1.9	588	10.7	9.9–11.5	2464	44.8	43.5–46.2	33	0.6	0.4–0.8

Note: * significantly higher than the total value; # significantly lower than the total value; *p* < 0.05 for all comparisons.

**Table 6:** Distribution of anti-HBs levels by HBV vaccination status.

Status	N	0–9.99 mIU/mL			10–50.99 mIU/mL			51–100.99 mIU/mL			101–149.99 mIU/mL			≥150 mIU/mL		
		*n*	%	95% CI	*n*	%	95% CI	*n*	%	95% CI	*n*	%	95% CI	*n*	%	95% CI
Vaccinated against HBV	3890	1859	47.8 #	46.2–49.4	625	16.1 *	14.9–17.3	315	8.1	7.3–9.0	164	4.2	3.6–4.9	927	23.8 *	22.5–25.2
Not vaccinated against HBV	1607	1174	73.1 *	70.8–75.2	139	8.6 #	7.4–10.1	54	3.4 #	2.6–4.4	42	2.6	1.9–3.5	198	12.3 #	10.8–14.0
Total	5497	3033	55.2	53.9–56.5	764	13.9	13.0–14.8	369	6.7	6.1–7.4	206	3.7	3.3–4.3	1125	20.5	19.4–21.6

Note: * significantly higher than the total value; # significantly lower than the total value; *p* < 0.05 for all comparisons.

**Table 7:** Serological markers of HBV infection by self-reported history (hepatitis B, vaccination).

Marker	HPNV				HPV				HNNV				HNV			
	N	*n*	%	95% CI	N	*n*	%	95% CI	N	*n*	%	95% CI	N	*n*	%	95% CI
HBsAg	34	10	29.4	16.8–46.2	16	2	12.5	3.5–36.0	1538	22	1.4	0.9–2.2	3839	45	1.2	0.9–1.6
Anti-HBc	34	25	73.5	56.9–85.4	16	10	62.5	38.6–81.5	1538	214	14	12.3–15.7	3839	322	8.4	7.6–9.3
Anti-HBs	34	12	35.3	21.5–52.1	16	8	50	28.0–72.0	1538	409	27	24.4–28.9	3839	2010	52.4	50.8–53.9

**Table 8:** Ratio of HBsAg-positive to occult hepatitis B forms by age group.

Age Group, Years	N	HBV DNA+, HBsAg+			HBV DNA+, HBsAg−		
		*n*	%	95% CI	*n*	%	95% CI
1–17	1415	2	0.1	0.0–0.5	14	1	0.5–1.7
1–5	369	1	0.3	0.0–1.5	5	1.4	0.4–3.1
6–11	510	0	0	0.0–0.7	3	0.6	0.1–1.7
12–17	536	1	0.2	0.0–1.0	6	1.1	0.4–2.4
18–29	796	7	0.9	0.4–1.8	2	0.3	0.0–0.9
30–39	838	7	0.8	0.3–1.7	10	1.2	0.6–2.2
40–49	914	12	1.3	0.7–2.3	5	0.5	0.2–1.3
50–59	900	14	1.6	0.9–2.6	1	0.1	0.0–0.6
60–69	930	15	1.6	0.9–2.6	6	0.6	0.2–1.4
70+	980	16	1.6	0.9–2.6	7	0.7	0.3–1.5
Total	6773	73	1.1	0.9–1.4	45	0.7	0.5–0.9

**Table 9:** Ratio of HBsAg-positive to occult hepatitis B forms by activity.

Field of Activity	N	HBV DNA+, HBsAg+			HBV DNA+, HBsAg−		
		*n*	%	95% CI	*n*	%	95% CI
Medicine	1366	13	1	0.5–1.6	7	0.5	0.2–1.1
Education	591	4	0.7	0.2–1.7	7	1.2	0.5–2.4
Preschooler	433	1	0.2	0.0–1.3	4	0.9	0.3–2.3
Schoolchildren	815	1	0.1	0.0–0.7	7	0.9	0.3–1.8
Students	213	2	0.9	0.1–3.4	1	0.5	0.0–2.6
Retirees	1156	22	1.9	1.2–2.9	7	0.6	0.2–1.2

**Table 10:** Ratio of HBsAg-positive to occult hepatitis B forms by iatrogenic intervention history.

History of Surgery or Blood Transfusion	N	HBV DNA+, HBsAg+			HBV DNA+, HBsAg−		
		*n*	%	95% CI	*n*	%	95% CI
Yes	2632	10	0.4 #	0.2–0.7	38	1.4	1.1–2.0
No	4141	63	1.5	1.2–1.9	7	0.2 #	0.1–0.3
Total	6773	73	1.1	0.9–1.4	45	0.7	0.5–0.9

Note: # significantly lower than the total value; *p* < 0.05 for all comparisons.

**Table 11:** Self-reported hepatitis B/C history by age group.

Age Group, Years	N	Experienced Hepatitis B			Experienced Hepatitis C		
		*n*	%	95% CI	*n*	%	95% CI
1–17	1415	0	0	0.0–0.3	1	0.1	0.0–0.4
1–5	369	0	0	0.0–1.0	0	0	0.0–1.0
6–11	510	0	0	0.0–0.7	1	0.2	0.0–1.1
12–17	536	0	0	0.0–0.7	0	0	0.0–0.7
18–29	796	0	0	0.0–0.5	1	0.1	0.0–0.7
30–39	838	5	0.6	0.3–1.4	1	0.1	0.0–0.7
40–49	914	12	1.3	0.8–2.3	7	0.8	0.4–1.6
50–59	900	12	1.3	0.8–2.3	4	0.4	0.2–1.1
60–69	930	14	1.5	0.9–2.5	8	0.9	0.4–1.7
70+	980	20	2 *	1.3–3.1	7	0.7	0.3–1.5
Total	6773	63	0.9	0.7–1.2	29	0.4	0.3–0.6

Note: * significantly higher than the total value; *p* < 0.05 for all comparisons.

**Table 12:** Hepatitis B markers by self-reported infectious history.

Self-Reported Infectious History	N	HBsAg			Anti-HBcAg			Anti-HBsAg			HBV DNA		
		*n*	%	95% CI	*n*	%	95% CI	*n*	%	95% CI	*n*	%	95% CI
Experienced hepatitis B	63	14	22.2 *	12.7–34.5	43	68.3 *	56.0–78.4	26	41.3	30.0–53.6	11	17.5 *	10.0–28.6
Never experienced hepatitis B	6559	94	1.4	1.2–1.8	694	10.6	9.9–11.3	2833	43.2	42.0–44.4	101	1.5	1.3–1.9
Total	6622	108	1.6	1.3–2.0	737	11.1	10.4–11.9	2859	43.2	42.0–44.4	112	1.7	1.4–2.0

Note: * significantly higher than the total value; *p* < 0.05 for all comparisons.

**Table 13:** Hepatitis C markers by self-reported infectious history.

Self-Reported Infectious History	N	Anti-HCV+			HCV RNA		
		*n*	%	95% CI	*n*	%	95% CI
Experienced hepatitis C	29	25	86.2 *	69.4–94.5	9	31 *	15.3–50.8
Never experienced hepatitis C	6610	95	1.4	1.2–1.8	10	0.2	0.1–0.3
Total	6639	120	1.8	1.5–2.2	19	0.3	0.2–0.5

Note: * significantly higher than the total value; *p* < 0.05 for all comparisons.

## Data Availability

The original contributions presented in this study are included in the article. Further inquiries can be directed to the corresponding author.

## Appendix A

**Table A1:** Viral markers (HB, HC, HD) by field of activity.

Field of Activity	N	HBsAg			Anti-HBcAg			Anti-HBsAg			Anti-HCV			Anti-HDV		
		*n*	%	95% CI	*n*	%	95% CI	*n*	%	95% CI	*n*	%	95% CI	*n*	%	95% CI
Medicine	1366	23	1.7	1.1–2.5	157	11.5	9.9–13.3	773	56.6 *	53.9–59.2	19	1.4	0.9–2.2	8	0.6	0.3–1.2
Science	117	1	0.9	0.0–4.7	12	10.3	6.0–17.1	53	45.3	36.6–54.3	1	0.9	0.2–4.7	0	0	0.0–1.1
Business	158	1	0.6	0.1–3.5	24	15.2	10.4–21.6	58	36.7	29.6–44.5	6	3.8	1.8–8.0	2	1.3	0.3–4.5
Education	592	10	1.7	0.8–3.1	71	12	9.6–14.9	243	41	37.2–45.1	12	2	1.2–3.5	2	0.3	0.1–1.2
Art/creativity	101	4	4	1.1–9.8	9	8.9	4.8–16.1	41	40.6	31.5–50.3	3	3	1.0–8.4	0	0	0.0–3.6
Production	269	6	2.2	0.8–4.8	40	14.9	11.1–19.6	115	42.8	37.0–48.7	2	0.7	0.2–2.7	1	0.4	0.1–2.1
Transport	100	0	0	0.0–3.6	4	4 #	1.6–9.8	37	37	28.2–46.8	4	4	1.6–9.8	0	0	0.0–3.6
Military service	19	0	0	0.0–17.6	1	5.3	0.9–24.6	7	36.8	19.1–59.0	0	0	0.0–17.6	0	0	0.0–17.6
Public service	242	4	1.7	0.5–4.2	21	8.7	5.7–12.9	99	40.9	34.9–47.2	3	1.2	0.4–3.6	0	0	0.0–1.5
Office	563	13	2.3	1.2–3.9	48	8.5	6.5–11.1	202	35.9 #	32.0–39.9	11	2	1.1–3.5	9	1.6	0.8–3.0
Unemployed	252	8	3.2	1.4–6.2	43	17.1 *	12.9–22.2	96	38.1	32.3–44.2	9	3.6	1.9–6.6	0	0	0.0–1.5
Preschooler	433	2	0.5	0.1–1.7	9	2.1 #	1.1–3.9	295	68.1 *	63.6–72.3	4	0.9	0.4–2.4	0	0	0.0–0.9
Miscellaneous	252	4	1.6	0.4–4.0	28	11.1	7.8–15.6	81	32.1 #	26.7–38.1	10	4	2.2–7.1	0	0	0.0–1.5
Schoolchild	815	1	0.1 #	0.0–0.7	25	3.1 #	2.1–4.5	343	42.1	38.7–45.5	8	1	0.5–1.9	3	0.4	0.1–1.1
Student	213	3	1.4	0.3–4.1	6	2.8 #	1.3–6.0	80	37.6	31.3–44.2	1	0.5	0.1–2.6	2	0.9	0.3–3.4
Retiree	1156	33	2.9	2.0–4.0	264	22.8 *	20.5–25.3	340	29.4 #	26.9–32.1	34	2.9	2.1–4.1	12	1	0.6–1.8
Tourism	18	0	0	0.0–18.5	1	5.6	1.0–25.8	8	44.4	24.6–66.3	0	0	0.0–18.5	0	0	0.0–18.5
Agriculture	23	2	8.7	1.1–28.0	3	13	4.5–32.1	8	34.8	18.8–55.1	0	0	0.0–14.8	0	0	0.0–14.8
IT	84	1	1.2	0.0–6.5	2	2.4 #	0.7–8.3	36	42.9	32.8–53.5	0	0	0.0–4.3	0	0	0.0–4.3
Total	6773	116	1.7	1.4–2.0	768	11.3	10.6–12.1	2915	43	41.9–44.2	127	1.9	1.6–2.2	39	0.6	0.4–0.8

Note: * significantly higher than the total value; # significantly lower than the total value; *p* < 0.05 for all comparisons.

**Table A2:** Distribution of hepatitis B serological profiles by activity group (corresponding to Figure 3).

Hepatitis B Markers	Medicine (N = 1366)			Education (N = 592)			Preschooler (N = 433)			Schoolchild (N = 815)			Student (N = 213)			Retiree (N = 1156)		
	*n*	%	95% CI	*n*	%	95% CI	*n*	%	95% CI	*n*	%	95% CI	*n*	%	95% CI	*n*	%	95% CI
HBsAg only	9	0.7	0.3–1.2	4	0.7	0.2–1.7	1	0.2	0.0–1.3	1	0.1	0.0–0.7	1	0.5	0.0–2.6	11	1	0.5–1.7
Anti-HBs only	627	45.9	43.2–48.6	185	31.3	27.5–35.2	288	66.5	61.9–70.9	325	39.9	36.5–43.3	74	34.7	28.4–41.5	151	13.1	11.2–15.1
Anti-HBc only	19	1.4	0.8–2.2	12	2	1.1–3.5	3	0.7	0.1–2.0	7	0.9	0.3–1.8	2	0.9	0.1–3.4	67	5.8	4.5–7.3
HBsAg, anti-HBs	10	0.7	0.4–1.3	2	0.3	0.0–1.2	1	0.2	0.0–1.3	0	0	0.0–0.5	2	0.9	0.1–3.4	4	0.3	0.1–0.9
HBsAg, anti-HBc	2	0.1	0.0–0.5	3	0.5	0.1–1.5	0	0	0.0–0.8	0	0	0.0–0.5	0	0	0.0–1.7	12	1	0.5–1.8
HBsAg, anti-HBs, anti-HBc	2	0.1	0.0–0.5	1	0.2	0.0–0.9	0	0	0.0–0.8	0	0	0.0–0.5	0	0	0.0–1.7	6	0.5	0.2–1.1
Anti-HBs, anti-HBc	134	9.8	8.3–11.5	55	9.3	7.1–11.9	6	1.4	0.5–3.0	18	2.2	1.3–3.5	4	1.9	0.5–4.7	179	15.5	13.4–17.7
Any of the following: HBsAg, anti-HBs, anti-HBc	803	58.8	56.1–61.4	262	44.3	40.2–48.4	299	69.1	64.5–73.4	351	43.1	39.6–46.5	83	39	32.4–45.9	430	37.2	34.4–40.1
The aforementioned markers are absent	563	41.2	38.6–43.9	330	55.7	51.6–59.8	134	30.9	26.6–35.5	464	56.9	53.5–60.4	130	61	54.1–67.6	726	62.8	59.9–65.6

**Table A3:** Distribution of hepatitis B serological profiles by history of iatrogenic interventions (surgery or blood transfusion).

Hepatitis B Markers	History of Surgery and/or Blood Transfusion (*n* = 2632)			No History of Surgery or Blood Transfusion (*n* = 4141)		
	*n*	%	95% CI	*n*	%	95% CI
HBsAg only	15	0.6	0.3–0.9	36	0.9	0.6–1.2
Anti-HBs only	772	29.3	27.6–31.1	1548	37.4	35.9–38.9
Anti-HBc only	76	2.9	2.3–3.6	93	2.3	1.8–2.7
HBsAg, anti-HBs	4	0.2	0.1–0.4	22	0.5	0.3–0.8
HBsAg, anti-HBc	10	0.4	0.2–0.7	20	0.5	0.3–0.7
HBsAg, anti-HBs, anti-HBc	2	0.1	0.0–0.3	7	0.2	0.1–0.3
Anti-HBs, anti-HBc	276	10.5	9.4–11.7	284	6.9	6.1- 7.7
Any of the following: HBsAg, anti-HBs, anti-HBc	1155	43.9	42.0–45.8	2010	48.5	47.0–50.1
The aforementioned markers are absent	1477	56.1	54.2–58.0	2131	51.5	49.9–53.0

**Table A4:** Anti-HBs levels in volunteers from St. Petersburg and the Leningrad region by age group.

Age Group, Years	N	Anti-HBs Levels, mIU/mL														
		0–9.99			10.0–50.99			51.0–100.99			101.0–149.99			≥150.0		
		*n*	%	95% CI	*n*	%	95% CI	*n*	%	95% CI	*n*	%	95% CI	*n*	%	95% CI
1–17	1415	717	50.7	48.1–53.3	266	18.8	16.8–20.9	118	8.3	7.0–9.9	62	4.4	3.4–5.6	252	17.8	15.9–19.9
1–5	369	111	30.1	25.6–34.9	77	20.9	17.0–25.3	37	10	7.4–13.5	23	6.2	4.2–9.2	121	32.8	28.2–37.7
6–11	510	254	49.8	45.5–54.1	110	21.6	18.2–25.3	45	8.8	6.7–11.6	26	5.1	3.5–7.4	75	14.7	11.9–18.0
12–17	536	352	65.7	61.6–69.6	79	14.7	12.0–18.0	36	6.7	4.9–9.2	13	2.4	1.4–4.1	56	10.4	8.1–13.3
18–29	796	319	40.1	36.7–43.5	140	17.6	15.1–20.4	62	7.8	6.1–9.9	40	5	3.7–6.8	235	29.5	26.5–32.8
30–39	838	374	44.6	41.3–48.0	130	15.5	13.2–18.1	63	7.5	5.9–9.5	47	5.6	4.2–7.4	224	26.7	23.8–29.8
40–49	914	551	60.3	57.1–63.4	100	10.9	9.1–13.1	48	5.3	4.0–6.9	24	2.6	1.8–3.9	191	20.9	18.4–23.7
50–59	900	595	66.1	63.0–69.1	91	10.1	8.3–12.3	33	3.7	2.6–5.1	25	2.8	1.9–4.1	156	17.3	15.0–19.9
60–69	930	653	70.2	67.2–73.1	86	9.2	7.5–11.3	41	4.4	3.3–5.9	26	2.8	1.9–4.1	124	13.3	11.3–15.7
70+	980	649	66.2	63.2–69.1	93	9.5	7.8–11.5	50	5.1	3.9–6.7	28	2.9	2.0–4.1	160	16.3	14.1–18.8
Total	6773	3858	57	55.8–58.1	906	13.4	12.6–14.2	415	6.1	5.6–6.7	252	3.7	3.3–4.2	1342	19.8	18.9–20.8

**Table A5:** Vaccination coverage (hepatitis B) by volunteer activity.

Field of Activity	N (Known History)	Vaccinated Against HBV		
		*n*	%	95% CI
Medicine	1208	999	82.7 *	80.5–84.7
Science	90	56	62.2	51.9–71.5
Business	115	56	48.7 #	39.8–57.7
Education	465	317	68.2	63.8–72.2
Art/creativity	65	40	61.5	49.4–72.4
Production	198	137	69.2	62.4–75.2
Transport	78	44	56.4	45.4–66.9
Military service	12	8	66.7	39.1–86.2
Public service	188	131	69.7	62.8–75.8
Office	429	248	57.8	53.1–62.4
Unemployed	195	130	66.7	59.8–72.9
Preschooler	415	380	91.6 *	88.5–93.9
Miscellaneous	186	120	64.5	57.4–71.0
Schoolchild	758	688	90.8 *	88.5–92.6
Student	188	166	88.3 *	82.9–92.1
Retiree	819	314	38.3 #	35.1–41.7
Tourism	11	8	72.7	43.4–90.3
Agriculture	17	9	52.9	31.0–73.8
IT	60	39	65	52.4–75.8
Total	5497	3890	70.8	69.5–72.0

Note: * significantly higher than the total value; # significantly lower than the total value; *p* < 0.05 for all comparisons.

**Table A6:** Distribution of participants by self-reported history of hepatitis B and vaccination status, stratified by age group.

Age Group, Years	N (Known History)	HPNV			HPV			HNNV			HNV		
		*n*	%	95% CI	*n*	%	95% CI	*n*	%	95% CI	*n*	%	95% CI
1–17	1328	0	0	0.0–0.3	0	0	0.0–0.3	121	9.1 #	7.7–10.8	1207	90.9 *	89.2–92.3
1–5	354	0	0	0.0–1.0	0	0	0.0–1.0	31	8.8 #	6.2–12.2	323	91.2 *	87.8–93.8
6–11	479	0	0	0.0–0.8	0	0	0.0–0.8	51	10.6 #	8.2–13.7	428	89.4 *	86.3–91.8
12–17	495	0	0	0.0–0.7	0	0	0.0–0.7	39	7.9 #	5.8–10.6	456	92.1 *	89.4–94.2
18–29	668	0	0	0.0–0.6	0	0	0.0–0.6	84	12.6 #	10.3–15.3	584	87.4 *	84.7–89.7
30–39	659	3	0.5	0.2–1.3	2	0.3	0.1–1.1	139	21.1 #	18.1–24.4	515	78.1 *	74.8–81.1
40–49	711	7	1	0.5–2.0	2	0.3	0.1–1.0	220	30.9	27.7–34.4	482	67.8	64.3–71.1
50–59	691	4	0.6	0.2–1.5	5	0.7	0.3–1.7	246	35.6 *	32.1–39.2	436	63.1 #	59.4–66.6
60–69	711	7	1	0.5–2.0	3	0.4	0.1–1.2	342	48.1 *	44.4–51.8	359	50.5 #	46.8–54.2
70+	659	13	2 *	1.2–3.3	4	0.6	0.2–1.6	386	58.6 *	54.8–62.3	256	38.8 #	35.2–42.6
Total	5427	34	0.6	0.4–0.9	16	0.3	0.2–0.5	1538	28.3	27.2–29.6	3839	70.7	69.5–71.9

Note: * significantly higher than the total value; # significantly lower than the total value; *p* < 0.05 for all comparisons.

**Table A7:** HBsAg detection frequency according to self-reported history of hepatitis B and vaccination status, by age group.

Age Group, Years	HPNV				HPV				HNNV				HNV			
	N	*n*	%	95% CI	N	*n*	%	95% CI	N	*n*	%	95% CI	N	*n*	%	95% CI
1–17	0	0	0	0.0–0.0	0	0	0	0.0–0.0	121	0	0	0.0–3.0	1207	3	0.2 #	0.1–0.7
1–5	0	0	0	0.0–0.0	0	0	0	0.0–0.0	31	0	0	0.0–11.2	323	2	0.6	0.1–2.2
6–11	0	0	0	0.0–0.0	0	0	0	0.0–0.0	51	0	0	0.0–7.0	428	0	0	0.0–0.9
12–17	0	0	0	0.0–0.0	0	0	0	0.0–0.0	39	0	0	0.0–9.0	456	1	0.2	0.0–1.2
18–29	0	0	0	0.0–0.0	0	0	0	0.0–0.0	84	2	2.4	0.3–8.3	584	8	1.4	0.6–2.7
30–39	3	0	0	0.0–70.8	2	0	0	0.0–84.2	139	2	1.4	0.2–5.1	515	8	1.6	0.7–3.0
40–49	7	2	28.6	8.2–64.1	2	0	0	0.0–84.2	220	2	0.9	0.1–3.2	482	11	2.3	1.1–4.0
50–59	4	2	50	15.0–85.0	5	1	20	3.6–62.4	246	3	1.2	0.3–3.5	436	4	0.9	0.3–2.3
60–69	7	2	28.6	8.2–64.1	3	0	0	0.0–70.8	342	5	1.5	0.5–3.4	359	7	1.9	0.8–4.0
70+	13	4	30.8	12.7–57.6	4	1	25	4.6–69.9	386	8	2.1	0.9–4.0	256	4	1.6	0.4–4.0
Total	34	10	29.4	16.8–46.2	16	2	12.5	3.5–36.0	1538	22	1.4	0.9–2.2	3839	45	1.2	0.9–1.6

Note: # significantly lower than the total value; *p* < 0.05 for all comparisons. Sample sizes per age group and category are identical to those shown in Table A6.

**Table A8:** Anti-HBc detection frequency according to self-reported history of hepatitis B and vaccination status, by age group.

Age Group, Years	HPNV				HPV				HNNV				HNV			
	N	*n*	%	95% CI	N	*n*	%	95% CI	N	*n*	%	95% CI	N	*n*	%	95% CI
1–17	0	0	0	–	0	0	0	–	121	4	3.3 #	1.3–8.2	1207	40	3.3 #	2.4–4.5
1–5	0	0	0	–	0	0	0	–	31	2	6.5	1.8–20.7	323	6	1.9 #	0.9–4.0
6–11	0	0	0	–	0	0	0	–	51	1	2 #	0.3–10.3	428	17	4 #	2.5–6.3
12–17	0	0	0	–	0	0	0	–	39	1	2.6	0.5–13.2	456	17	3.7 #	2.3–5.9
18–29	0	0	0	–	0	0	0	–	84	0	0 #	0.0–4.3	584	13	2.2 #	1.3–3.8
30–39	3	3	100	43.8–100.0	2	1	50	9.5–90.5	139	6	4.3 #	2.0–9.1	515	26	5 #	3.5–7.3
40–49	7	5	71.4	35.9–91.8	2	2	100	34.2–100.0	220	20	9.1	6.0–13.6	482	53	11	8.5–14.1
50–59	4	3	75	30.1–95.4	5	4	80	37.6–96.4	246	31	13	9.0–17.3	436	65	14.9 *	11.9–18.6
60–69	7	5	71.4	35.9–91.8	3	1	33.3	6.1–79.2	342	56	16	12.8–20.7	359	64	17.8 *	14.2–22.1
70+	13	9	69.2	42.4–87.3	4	2	50	15.0–85.0	386	97	25 *	21.1–29.7	256	61	23.8 *	19.0–29.4
Total	34	25	73.5	56.9–85.4	16	10	62.5	38.6–81.5	1538	214	14	12.3–15.7	3839	322	8.4	7.6–9.3

Note: * significantly higher than the total value; # significantly lower than the total value; *p* < 0.05 for all comparisons. Sample sizes per age group and category are identical to those shown in Table A6.

**Table A9:** Anti-HBs detection frequency according to self-reported history of hepatitis B and vaccination status, by age group.

Age Group, Years	HPNV				HPV				HNNV				HNV			
	N	*n*	%	95% CI	N	*n*	%	95% CI	N	*n*	%	95% CI	N	*n*	%	95% CI
1–17	0	0	0	–	0	0	0	–	121	36	30	22.3–38.4	1207	624	51.7	48.9–54.5
1–5	0	0	0	–	0	0	0	–	31	15	48 *	32.0–65.2	323	233	72.1 *	67.0–76.7
6–11	0	0	0	–	0	0	0	–	51	15	29	18.7–43.0	428	227	53	48.3–57.7
12–17	0	0	0	–	0	0	0	–	39	6	15	7.2–29.7	456	164	36 #	31.7–40.5
18–29	0	0	0	–	0	0	0	–	84	41	49 *	38.4–59.3	584	362	62 *	58.0–65.8
30–39	3	2	66.7	20.8–93.9	2	0	0	0.0–84.2	139	47	34	26.5–42.0	515	327	63.5 *	59.3–67.5
40–49	7	3	42.9	15.8–75.0	2	2	100	34.2–100.0	220	47	21	16.5–27.2	482	246	51	46.6–55.5
50–59	4	1	25	4.6–69.9	5	3	60	23.1–88.2	246	49	20	15.4–25.4	436	206	47.2	42.6–51.9
60–69	7	2	28.6	8.2–64.1	3	1	33.3	6.1–79.2	342	75	22	17.9–26.6	359	142	39.6 #	34.6–44.7
70+	13	4	30.8	12.7–57.6	4	2	50	15.0–85.0	386	114	30	25.2–34.3	256	103	40.2 #	34.4–46.3
Total	34	12	35.3	21.5–52.1	16	8	50	28.0–72.0	1538	409	27	24.4–28.9	3839	2010	52.4	50.8–53.9

Note: * significantly higher than the total value; # significantly lower than the total value; *p* < 0.05 for all comparisons. Sample sizes per age group and category are identical to those shown in Table A6.

**Table A10:** Distribution of hepatitis B serological profiles by self-reported history of hepatitis B and vaccination status (HPNV, HPV, HNNV, HNV).

Hepatitis B Markers	HPNV (*n* = 34)			HPV (*n* = 16)			HNNV (*n* = 1538)			HNV (*n* = 3839)		
	*n*	%	95% CI	*n*	%	95% CI	*n*	%	95% CI	*n*	%	95% CI
HBsAg only	3	8.8	3.0–23.0	1	6.3	1.1–28.3	10	0.7	0.3–1.2	18	0.5	0.3–0.7
Anti-HBs only	0	0	0.0–0.0	1	6.3	1.1–28.3	251	16.3	14.5–18.3	1733	45.1	43.6–46.7
Anti-HBc only	6	17.6	8.3–33.5	2	12.5	3.5–36.0	55	3.6	2.7–4.6	56	1.5	1.1–1.9
HBsAg, anti-HBs	0	0	0.0–0.0	0	0	0.0–0.0	4	0.3	0.1–0.7	17	0.4	0.3–0.7
HBsAg, anti-HBc	7	20.6	10.3–36.8	1	6.3	1.1–28.3	5	0.3	0.1–0.8	6	0.2	0.1–0.3
HBsAg, anti-HBs, anti-HBc	0	0	0.0–0.0	0	0	0.0–0.0	3	0.2	0.0–0.6	4	0.1	0.0–0.3
Anti-HBs, anti-HBc	12	35.3	21.5–52.1	7	43.8	23.1–66.8	151	9.8	8.4–11.4	256	6.7	5.9–7.5
Any of the following: HBsAg, anti-HBs, anti-HBc	28	82.4	66.5–91.7	12	75	50.5–89.8	479	31.1	28.8–33.5	2090	54.4	52.8–56.0
The aforementioned markers are absent	6	17.6	8.3–33.5	4	25	10.2–49.5	1059	68.9	66.5–71.2	1749	45.6	44.0–47.2

Note: Sample sizes per age group and category are identical to those shown in Table A6.
